# Automatic imitation of speech is enhanced for non-native sounds

**DOI:** 10.3758/s13423-023-02394-z

**Published:** 2023-10-17

**Authors:** Hannah Wilt, Yuchunzi Wu, Bronwen G. Evans, Patti Adank

**Affiliations:** 1 Department of Speech, Hearing and Phonetic Sciences, University College London, London, UK; 2 Department of Neural and Cognitive Sciences, New York University Shanghai, Shanghai, China; 3 NYU-ECNU Institute of Brain and Cognitive Sciences at New York University Shanghai, Shanghai, China

**Keywords:** Automatic imitation, Second language processing, Speech, Stimulus response compatibility, Non-native speech perception

## Abstract

Simulation accounts of speech perception posit that speech is covertly imitated to support perception in a top-down manner. Behaviourally, covert imitation is measured through the stimulus-response compatibility (SRC) task. In each trial of a speech SRC task, participants produce a target speech sound whilst perceiving a speech distractor that either matches the target (compatible condition) or does not (incompatible condition). The degree to which the distractor is covertly imitated is captured by the *automatic imitation effect*, computed as the difference in response times (RTs) between compatible and incompatible trials. Simulation accounts disagree on whether covert imitation is enhanced when speech perception is challenging or instead when the speech signal is most familiar to the speaker. To test these accounts, we conducted three experiments in which participants completed SRC tasks with native and non-native sounds. Experiment 1 uncovered larger automatic imitation effects in an SRC task with non-native sounds than with native sounds. Experiment 2 replicated the finding online, demonstrating its robustness and the applicability of speech SRC tasks online. Experiment 3 intermixed native and non-native sounds within a single SRC task to disentangle effects of perceiving non-native sounds from confounding effects of producing non-native speech actions. This last experiment confirmed that automatic imitation is enhanced for non-native speech distractors, supporting a compensatory function of covert imitation in speech perception. The experiment also uncovered a separate effect of producing non-native speech actions on enhancing automatic imitation effects.

**Supplementary Information:**

The online version contains supplementary material available at 10.3758/s13423-023-02394-z.

## Introduction

Action observation engages neural mechanisms of action execution (Buccino et al., 2001; Fadiga et al., 1995; Nishitani & Hari, 2002). For vocal actions, the engagement of speech-production mechanisms in speech perception has been demonstrated using functional magnetic resonance imaging (fMRI) (Park, 2020; Pulvermüller et al., 2006; Wilson et al., 2004), transcranial magnetic stimulation (TMS) (Fadiga et al., 2002; Murakami et al., 2011; Watkins et al., 2003), and electroencephalography (EEG) (Michaelis et al., 2021; Oliveira et al., 2021; Pastore et al., 2022). Simulation accounts of speech perception (Pickering & Garrod, 2013; Wilson & Knoblich, 2005) propose that speech actions are automatically and covertly imitated by listeners. This covert imitative process informs forward models of the perceived speech, conducting real-time simulations to generate top-down predictions of the speech signal to support perception.

Evidence for a causal role of covert imitation in speech perception comes from experiments using TMS to temporarily disrupt speech motor areas. D’Ausilio et al. (2009) found that inhibitory stimulation of the lip area of the primary motor cortex (M1) specifically hindered discrimination of lip-articulated contrasts, while stimulation of tongue M1 obstructed discrimination of tongue sounds. Möttönen and Watkins (2009) showed that inhibitory TMS to lip M1 disrupted participants’ phonemic categorisation of lip-articulated speech sounds. The articulator-specific disruption of phonetic perception through inhibitory stimulation of motor areas supports a role for covert imitation in speech perception.

Behaviourally, covert imitation is measured through stimulus-response compatibility (SRC) paradigms. In manual SRC tasks (e.g. Brass et al. 2000), participants perform an action prompted by a visual cue (e.g., index-finger movement prompted by a “1”) while a distractor is presented. The distractor is compatible (e.g., video clip of the same index-finger movement) or incompatible with the target response (e.g., video clip of a middle-finger movement). Slower response times (RTs) for incompatible target-distractor pairs compared to compatible pairs are thought to reflect the automatic activation of motor processes elicited by the distractor, facilitating responses for compatible trials and inhibiting responses for incompatible trials (Heyes, 2011). The *automatic imitation effect*, computed as the difference in RTs between incompatible and compatible trials, indexes covert imitation of the distractor stimulus. In speech SRC tasks, participants produce speech sounds in response to prompts superimposed over a distractor (e.g., a video of a speaker saying [ba]). Using auditory-only, visual-only or audiovisual distractors, speech SRC tasks have demonstrated significant automatic imitation effects for consonants (Galantucci et al., 2009; Ghaffarvand Mokari et al., 2020; Jarick & Jones, 2009; Kerzel & Bekkering, 2000; Roon & Gafos, 2015; Trotter et al., 2023; Wilt et al., 2022; Wu et al., 2019) and vowels (Adank et al. 2018; Ghaffarvand Mokari et al. 2020; Ghaffarvand Mokari et al. 2021).

Motor activation during speech perception has been demonstrated extensively for sounds in the perceiver’s native repertoire, yet the implication of covert imitation in processing unfamiliar speech sounds is less well established. Simulation theories disagree on the conditions under which covert imitation occurs, leading to distinct predictions on the implication of covert imitation in non-native speech perception. Wilson and Knoblich (2005) propose that imitative motor activation serves as a compensatory mechanism when speech perception is challenging, as is the case when processing non-native speech sounds (Adank et al., 2009; Floccia et al., 2009; van Wijngaarden, 2001). Hence, this account predicts that perceiving non-native sounds elicits more covert imitation than native sounds. Alternatively, Pickering and Garrod’s integrated theory of language production and comprehension (Pickering & Garrod, 2013) posits that speech perception preferably relies on covert imitation when the signal is familiar to the listener, utilising the “simulation route” for action perception. When the speech is unfamiliar, speech perception relies more on auditory mechanisms (the “association route”). Covert imitation is expected to be enhanced when listening to native speech sounds compared to non-native speech sounds (Pickering & Gambi, 2018). Predictions of the integrated theory of language are consistent with theories of action perception claiming that action-perception associations are learned through sensorimotor experience, for example, the Theory of Event Coding (Hommel, 2009, 2019) and Associative Sequence Learning (Heyes, 2005, 2011).

Wilson and Knoblich’s proposal of a compensatory role of covert imitation in speech perception is supported by evidence of enhanced motor activity during the perception of motor and noise-distorted speech compared to clear speech (Alain et al., 2018; Du et al., 2016; Nuttall et al., 2016, 2017). In a transcranial direct current stimulation (tDCS) study (Sehm et al., 2013), facilitatory stimulation of the left inferior frontal gyrus (IFG) enhanced perceptual learning of degraded speech with low intelligibility, suggesting that speech production areas support perception under challenging listening conditions. Enhanced motor recruitment during non-native versus native speech processing has been reported in several fMRI studies (Callan et al., 2003, 2004, 2014; Golestani, 2016; Wilson & Iacoboni, 2006) and TMS experiments (Schmitz et al., 2019), though the opposite effect has also been observed for visual-only speech videos (Swaminathan et al., 2013). Further, infant studies have highlighted a role for production processes in perceiving novel speech sounds. An MEG study by Kuhl et al. (2014) found that while 7-month-old infants displayed comparable activation of auditory and motor cortices when listening to native and non-native speech, by 11–12 months activation was greater in motor regions for non-native speech. Bruderer et al. (2015) demonstrated that pre-verbal infants’ auditory discrimination of the Hindi [d̪]–[ɖ] contrast was hindered by teethers restraining tongue movements, but not by teethers that did not restrict tongue mobility. Together, these studies suggest that speech-production mechanisms may be preferentially activated for unfamiliar sounds.

In contrast, Pickering and Garrod’s proposition of enhanced covert imitation during perception of familiar speech actions aligns with the literature on the covert imitation of manual and bodily actions. Neuroimaging studies have reported enhanced motor activation with increasing familiarity to perceived movements (Calvo-Merino et al., 2005; Haslinger et al., 2005; Margulis et al., 2009), though the opposite effect has also been reported (Liew et al., 2011). Behaviourally in manual SRC tasks, automatic imitation effects increase following mirror training (e.g., participants close their hand when seeing a video of a hand closing) and disappear following counter-mirror training (e.g., participants open their hand when seeing a video of a hand closing) (Cook et al., 2010; Gillmeister et al., 2008; Heyes et al., 2005). In a similar study using speech stimuli (Wu et al., 2019), automatic imitation significantly increased following mirror training (participants produced the same syllable as that presented in audiovisual stimuli) and decreased non-significantly following counter-mirror training (participants produced the alternative syllable to that presented in audiovisual stimuli). Taken together, this line of evidence suggests that covert imitation is enhanced by familiarity and experience.

We aimed to test predictions from Wilson and Knoblich and from Pickering and Garrod in three speech SRC experiments. These experiments aimed to establish whether automatic imitation effects evoked by unfamiliar, non-native speech sounds are greater (as predicted by Wilson and Knoblich) or smaller (as predicted by Pickering and Garrod) than automatic imitation elicited by familiar, native speech sounds. In Experiment 1, participants completed an SRC task with native sounds and an SRC task with non-native sounds. Experiment 2 replicated Experiment 1 online, to strengthen our findings through replication (Schmidt, 2009) and to validate that speech SRC tasks can successfully be conducted online (Wilt et al., 2022). In Experiment 3, participants produced and perceived native and non-native sounds within a single SRC task, allowing to disentangle effects of perceiving non-native distractors from potential effects of producing unfamiliar speech actions on automatic imitation.

## Experiment 1

### Methods

#### Participants

Sixty-five participants were recruited. All self-reported being native British-English speakers with normal hearing, normal or corrected-to-normal vision and no speech disorders or neurological disorders. Participants received £20 compensation or course credit for this experiment, which constituted the pre-training session of a two-part study cut short by COVID-19. Sixteen participants were excluded: seven participants did not follow task instructions; one did not complete the full two-part study; one spoke Welsh and hence was familiar to the non-native sound [ɮɑ]; five participants had error rates (ERs) of > 50% in one or more of the SRC tasks; one had an overall ER of over three standard deviations (SDs) from the group mean; one was excluded due to a software error. The final sample comprised 49 participants (33 female, *M*_age_ = 23.41 years). The full list of languages spoken by the participants is available in Online Supplemental Material (OSM) Appendix A.

#### Stimuli

**Fig. 1:**
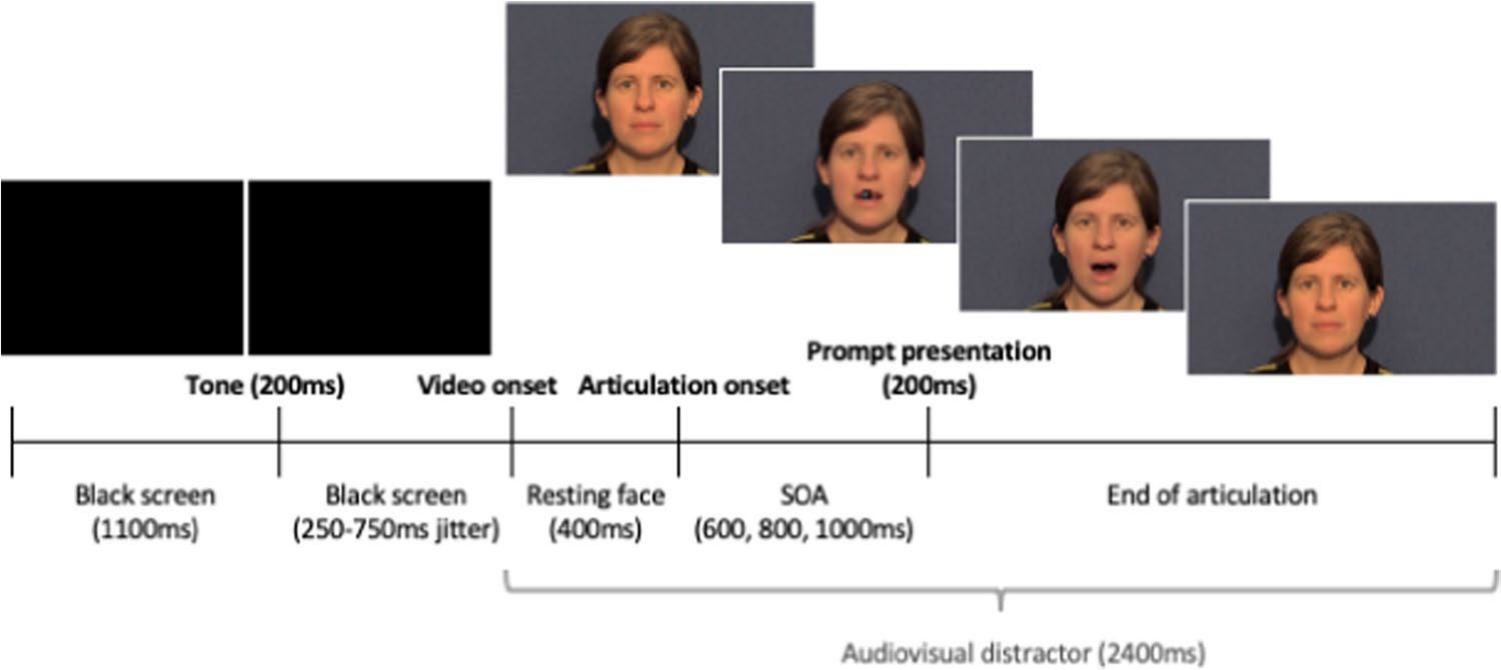
Stimulus-response compatibility (SRC) trial timeline for Experiment 1. Timings in parentheses represent durations

Videos showed a phonetically trained female native British-English speaker from the neckline upward over a blue background. The videos were filmed using a Canon Legria video camera and edited in iMovie and MATLAB. Each video lasted 2,400 ms, beginning and ending with the speaker in resting configuration. The auditory stimuli consisted of productions of [bɑ] (voiced bilabial plosive), [lɑ] (voiced alveolar lateral approximant), [ʙɑ] (voiced bilabial trill) and [ɮɑ] (voiced alveolar lateral fricative) by the same female speaker, recorded using a RØDE NT1-A Condenser Microphone and root-mean-square normalised on Praat (Boersma & Weenink, 2018). The non-native sounds [ʙɑ] and [ɮɑ] were selected as these were both visually and auditorily distinct from one another and from any British English sounds, and hence recognisable to British English speakers without perceptual training. Video and auditory stimuli were aligned on Presentation to create the distractor videos. Key articulatory event timings are displayed in OSM Appendix B. Response prompts comprised of the symbols £, %, &, # in white Helvetica font size 36 pt on a black background, superimposed over the distractor videos using Presentation. These appeared over the speaker’s lips at one of three stimulus onset asynchronies (SOAs): 600 ms, 800 ms or 1,000 ms post articulation onset. The utilisation of multiple SOAs is standard practice in SRC studies to examine the time course of effects. Distractor videos were preceded by a 1,100-ms black screen, followed by a 500-Hz tone for 200 ms after which the screen remained black for an additional jittered duration of 250, 375, 500, 652 or 750 ms (Fig. 1).

Production instruction videos were recorded for each of the four speech sounds. The same female speaker was presented from the neckline upward in front of the blue background. In each video, the speaker first produced the speech sound, followed by an oral description of how to produce the sound, and finally two more productions of the sound. For the [bɑ] sound, instructions were *“To produce this sound, bring your lips together to block airflow, let the air out in one burst, and say /a/”.* For the [lɑ] sound, instructions were *“To produce this sound, move your lips apart slightly, place the tip of your tongue behind your upper teeth to block airflow, release the air slowly, letting it pass by the sides of your tongue, and say /a/”.* For the [ʙɑ] sound, instructions were “*To produce this sound, bring your lips together to block airflow, release the air slowly, letting it pass between your lips, as if to blow raspberries, and say /a/”.* For the [ɮɑ] sound, instructions were *“To produce this sound, move your lips apart slightly, place the tip of your tongue behind your upper teeth to block airflow, raise the sides of your tongue, release the air slowly, causing turbulence and letting it pass over the sides of your tongue, and say /a/”.*

#### Procedure

The experiment was conducted in a soundproofed, light-controlled booth. Participants wore a Beyerdynamic DT 297 PV MK II headset as they completed two SRC tasks in Presentation on a Dell PC. In the native SRC task, responses and distractors were [bɑ] and [lɑ]. In the non-native SRC task, responses and distractors were [ʙɑ] and [ɮɑ]. Before each SRC task, speech production instruction videos were displayed on the screen for the two relevant sounds. Participants could play the videos as many times as they wanted and were asked to produce each sound at least five times and/or until the researcher was satisfied with their production. Next, participants learned the prompt-response pairings for each speech sound in the task. Symbols were displayed on the screen above videos of the speaker producing the associated sounds. Twenty-four possible prompt-response pairings where created, to which participants were randomly assigned.

For the SRC tasks, participants were instructed to produce the sound prompted by the symbol cue as quickly as possible and to ignore the distractor video. For each task (native and non-native), participants first completed 20 randomly selected practice trials, followed by six blocks of 30 trials each (180 trials total per task). The order in which the native and non-native SRC tasks were performed was randomised and counterbalanced across participants. Altogether, the testing session lasted approximately 50 min.

#### Data processing and analysis

Participants’ vocal responses were recorded using a Beyerdynamic DT 297 PV MK II headset microphone. Recordings started at video onset for 3,000 ms. Response annotations and RT measurements were manually determined on Praat. Errors were defined as productions of the wrong or of multiple responses, missing answers or anticipatory responses with RTs < 200 ms. For the non-native sounds, productions were considered erroneous if they could not be clearly auditorily identified as attempts to produce either [ʙɑ] or [ɮɑ], and if the spectrogram did not show clear turbulence (for [ʙɑ] and [ɮɑ]) and/or at least one vocal tract resonance portion (for [ʙɑ] only) (see Kavitskaya et al., 2009).

For the 49 participants, 18,000 observations were collected. Erroneous trials were removed from the analyses (914, 5.08%): 829 productions of the wrong or of multiple prompts; 41 missing answers; and 44 anticipatory responses. Error rates (ERs) averaged 3.96% (*SD* = 5.02%) in the native task and 6.20% (*SD* = 7.11%) in the non-native task. A further 1,380 trials were excluded in which RTs surpassed three median absolute deviations (MADs) from a participant’s mean RT for each experimental condition. The remaining 15,706 trials were included in the analyses.

Raw RTs for correct trials were analysed with general linear mixed effects models in R using the *lme4* package in R (Bates et al., 2014). Fixed factors were Nativeness (native vs. non-native), Compatibility (compatible vs. incompatible), SOA (SOA1 (600 ms), SOA2 (800 ms), SOA3 (1,000 ms)) and their interactions. Nativeness was coded as -0.5 and 0.5 for the native and non-native conditions, respectively, and Compatibility was coded as -0.5 and 0.5 for compatible and incompatible trials, respectively. This coding scheme is considered preferable to treatment coding in modelling interactions (Singmann & Kellen, 2019). As we were interested in the successive effects of SOA, backward difference coding was used for this factor, allowing for sequential comparisons between each level and its immediate preceding level (i.e., SOA2 vs. SOA1, SOA3 vs. SOA2).

We assumed a gamma distribution and identity link function following Lo and Andrews (2015). This type of link function is considered preferable to transformation for RT data (Balota et al., 2013; Lo & Andrews, 2015; Schramm & Rouder, 2019) and allowed us to avoid potential issues reported with log-transforming and subsequently back transforming RT data (Feng et al., 2013; Lo & Andrews, 2015; Manandhar & Nandram, 2021; Molina & Martín, 2018). Following Barr et al. (2013), the maximal random effect structure to converge and pass singularity checks was used. This included by-participant random intercepts and slopes for Nativeness. Backward selection was then used to identify the model that best fit the dataset. Starting with higher order interactions, predictors were removed systematically and chi-squared tests performed using *anova().* Fixed factors were removed from the final model if they did not significantly benefit model fit (*p* > .05) and were not included in any higher order interactions. At each step, the factor for which there was least evidence of inclusion (i.e., the highest p-value in the chi-squared test) was removed first and the remaining factors reassessed. We stopped when there were no more fixed factors to remove, i.e., when all remaining factors either significantly improved model fit or were included in significant higher-order interactions.

### Results

**Fig. 2:**
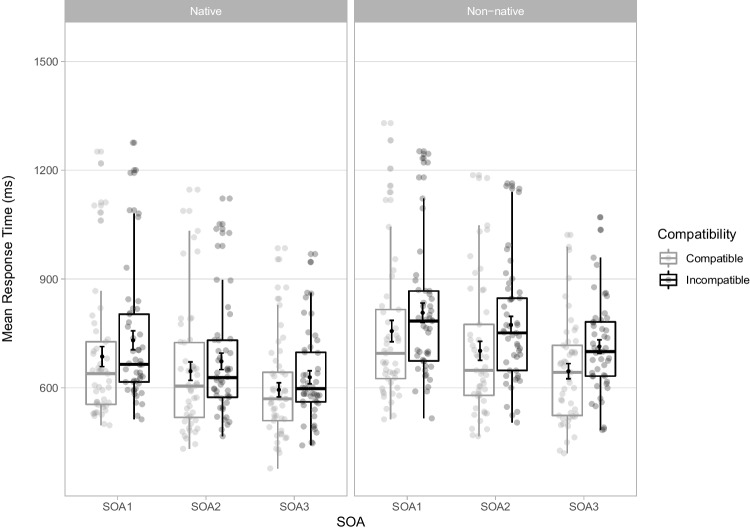
Mean response times (RTs) in milliseconds (ms) for correct trials in the stimulus-response compatibility (SRC) tasks for each experimental condition in Experiment 1. Points in the background show the raw mean RTs for each participant (points are offset on the x-axis for clarity). The boxplots indicate the first, second (median) and third quartiles, and whiskers indicate 1.5 times the interquartile range of the distribution. Black points in the foreground show the mean and error bars indicate standard errors

In all, 15,706 trials were analysed. Mean RTs for each experimental condition are displayed in Fig. 2 and OSM Appendix C. All main effects and interactions were included in the final model (Table 1), as the three-way interaction Compatibility x Nativeness x SOA significantly improved model fit (χ^2^(2) = 11.855, *p* = .003).

**Table 1:** Final model of raw reaction times (RTs) in milliseconds (ms) using a gamma distribution and identity link function for Experiment 1

Fixed effect	Estimate	*SE*	t-value	*p*-value
(Intercept)	**736**	**3**	**279.095**	**< 2 x 10**^-16^***
Nativeness	**72**	**3**	**24.727**	**< 2 x 10**^-16^***
Compatibility	**48**	**3**	**18.977**	**< 2 x 10**^-16^***
SOA2-1	**-51**	**3**	**-16.981**	**< 2 x 10**^-16^***
SOA3-2	**-59**	**2**	**-26.010**	**< 2 x 10**^-16^***
Nativeness x Compatibility	**28**	**4**	**7.383**	**1.55 x 10**^-13^***
Nativeness x SOA2-1	5	4	1.487	0.137
Nativeness x SOA3-2	**-10**	**2**	**-4.228**	**2.36 x 10**^-5^***
Compatibility x SOA2-1	4	3	1.361	0.174
Compatibility x SOA3-2	2	3	0.771	0.441
Nativeness x Compatibility x SOA2-1	**31**	**3**	**10.210**	**< 2 x 10**^-16^***
Nativeness x Compatibility x SOA3-2	-6	3	-1.821	0.069

*Note:* SOA = stimulus-onset asynchrony. Bold entries indicate significant effects. * *p* < .05, ** *p* < .01, *** *p* < .001

There was a significant main effect of Compatibility, with slower RTs in incompatible (*M* = 721 ms, *SD* = 170 ms) than compatible trials (*M* = 672 ms, *SD* = 181 ms). The overall automatic imitation effect averaged 49 ms (*SD* = 63 ms), computed from aggregated RTs per participant and experimental condition. The main effect of Nativeness was significant with slower RTs in the non-native (*M* = 733 ms, *SD* = 177 ms) than the native SRC task (*M* = 660 ms, *SD* = 167 ms). The significant main effect of SOA demonstrated that RTs decreased from SOA1 (*M* = 745 ms, *SD* = 196 ms) to SOA2 (*M* = 698 ms, *SD* = 176 ms) to SOA3 (*M* = 646 ms, *SD* = 141 ms). The interaction Nativeness x Compatibility was significant, with larger automatic imitation effects for non-native (*M* = 63 ms, *SD* = 72 ms) than for native sounds (*M* = 36 ms, *SD* = 49 ms). This interaction was modulated by SOA, as the difference in automatic imitation effects between native and non-native tasks was smaller at SOA1 (6 ms) than SOA2 (45 ms) and SOA3 (33 ms).

Experiment 1 uncovered enhanced automatic imitation for non-native sounds, in line with Wilson and Knoblich’s account of a compensatory role of covert imitation in speech perception.

## Experiment 2

### Methods

#### Participants

One hundred and eighty-four participants were recruited for the eligibility screening. All were self-reported monolingual native British-English speakers, with normal hearing, normal or corrected-to-normal vision, and no speech or neurological disorders. Recruitment was conducted on Prolific (/prolific.co) and testing on Gorilla (/gorilla.sc). Participants were required to run the study on a computer and through Chrome, using wired headphones and microphones that were not inbuilt to the computer. Of the 184 participants who completed the eligibility screening, 65 were invited to take part in the main experiment. A further 26 were excluded from the analyses, see exclusions detailed in Table 2. The final sample consisted of 39 participants (25 female, *M*_age_ = 25 years). Participants received £0.50 for the eligibility screening and £5.50 for the main task, commensurate to £7.50 per hour.

**Table 2:** Exclusion numbers and criteria at each stage of Experiment 2

Experiment stage	Starting *n*	Exclusion criteria	*n* exclusions
Eligibility screening	184	Did not meet inclusion criteria	**119** (64.67%)
Main study	65	Did not start the main experiment	2
		Failed the headphone check	6
		Failed the catch trials	0
		Did not finish the experiment	4
		Total exclusions	**12** (18.46%)
Data processing	53	Did not follow task instructions	4
		Low quality audio	5
		High variability in distractor onsets	4
		Error rate >3 SD from group mean	1
		Total exclusions	**14** (26.42%)

*Note. n* = participant number. Bold entries indicate total exclusions at the end of each stage. Final *n* = 39

#### Stimuli

**Fig. 3:**
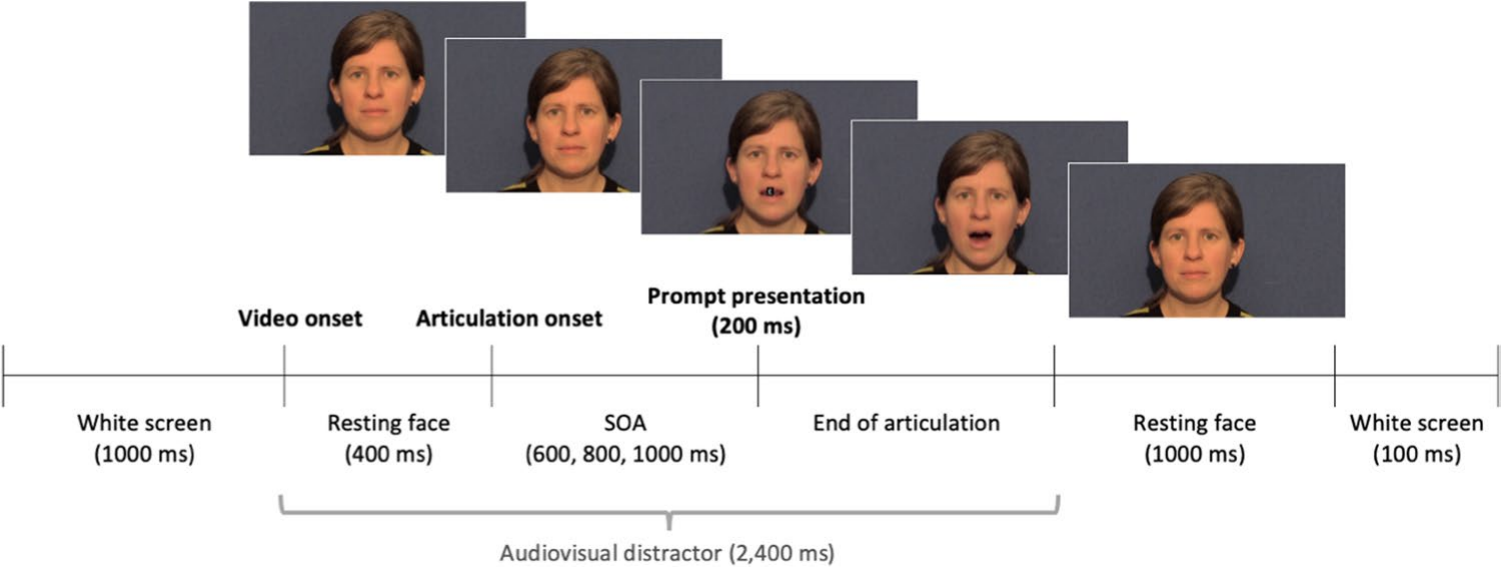
Stimulus-response compatibility (SRC) trial timeline for Experiment 2. Timings in parentheses represent durations

The video and audio recordings were the same as that used in Experiment 1. Audiovisual stimuli were created by aligning video, auditory and prompt stimuli on Shotcut (/shotcut.org) and saving the files in MP4 format. This was preferred over overlaying the elements on Gorilla in order to avoid stimuli onset lags (Bridges et al., 2020) and to ensure precision in prompt onsets, (cf. Wilt et al., 2022). Symbol prompts £, %, &, # appeared in white Arial font size 50 pt on a black background over the speaker’s lips at one of the three SOAs (600, 800 or 1,000 ms post articulation onset). Separate videos were created for each combination of speech sound and SOA as well as for each of four prompt-response pairing counterbalances. On Gorilla, the distractor videos were preceded by a white screen for 1,000 ms (Fig. 3). After the 2,400-ms video was presented, the female speaker remained in resting position for 1,100 ms before the onset of the next trial. Production instruction videos were identical to that used in Experiment 1.

Tone stimuli for the eligibility screening consisted of three 200-ms synthetic periodic tones at 200, 300 and 440 Hz. Tone stimuli for the catch trials were 200-ms synthetic period tones at 350 Hz generated on Praat. Separate sound files were created for each of the three conditions (one, two and three tones), with 32-ms silence between tones in multi-tone conditions. The headphone check stimuli consisted of sequences of three 200 Hz sine wave tones playing for 1,000 ms each, one at -20dB and two at -14dB.

#### Procedure

Participants first completed an eligibility screening test on Gorilla to assess the quality and consistency of the recordings obtained with their hardware. Participants were instructed to place their headset next to their microphone, to turn their sound to maximum and make as little noise as possible throughout the trials. In each 4100ms trial, the instructions “*Please wait patiently. You do not need to do anything for this task”* were displayed over a white background and a 200-ms tone played at 1,500 ms. Each of three pure tones was played twice, rendering six trials total. Recordings were set to start at 500 ms for 3,400 ms. The procedure lasted approximately 2 min. Participants were considered eligible if a minimum of five out of six recordings picked up the tone stimuli, were systematically longer than 2,500 ms, clear of static noise, and if tone onsets in the recordings were within a 50-ms range.

Eligible participants were invited to partake in the main experiment and asked to complete the experiment within 48 h of receiving their eligibility status. Participants were instructed to complete the study in a quiet room by themselves, with as little distractions as possible and using the same hardware as in the eligibility screening. Participants first completed a headphone screening test in which they had to judge which of three pure tones was the quietest (Woods et al., 2017; Wilt et al., 2022). A minimum of four out of six correct responses was required to access the main experiment and receive remuneration.

In the main experiment, participants completed an SRC task with native sounds and an SRC task with non-native sounds. Before each task, participants viewed SRC task instructions as well as the relevant prompt-response pairings. Pairings were presented through displays in which the two relevant symbols were arranged on either side of the screen and participants could press a ‘*play’* button under each symbol to hear the associated sound up to three times. Next, participants viewed each production instruction videos twice and were asked to produce each sound at least five times. Two sample trials were then presented to participants, as well as a reminder of the prompt-response pairings.

For the main SRC task, participants completed 12 practice trials (2 distractors x 2 compatibility conditions x 3 SOAs), followed by six blocks of 24 trials (144 trials total per task). Before each new block, participants were reminded of the prompt-response pairings in displays allowing each sound to be played once. The order in which the native and non-native SRC tasks were completed was randomised and counterbalanced across participants in a Latin square design, as was assignment to one of four possible prompt-response pairings.

To check that participants were not muting the experiment, a catch trial was included randomly in each of the six blocks (Wilt et al. 2022). In the catch trials, the screen remained white as a rapid succession of one to three synthetic periodic tones played. Participants indicated via response button how many tones they heard (one, two or three). A performance below four out of six in the catch trials of each SRC task (chance performance: two out of six) would result in the automatic rejection of the participant from the experiment. No participants met this exclusion criteria.

After completing the native and non-native SRC tasks, participants performed a video-onset detection task (Wilt et al., 2022). This task was included to estimate latencies between recording onsets and SRC video onsets, to adjust RT obtained in the main SRC task. The same instructions were provided as in the eligibility screening test, i.e., participants were to place their headphones close to their microphone and turn their system volume to maximum to obtain recordings of the stimuli. Instead of tone stimuli as in the eligibility screening, the SRC video stimuli were played and recorded. Recordings were set to start with video stimuli onset and last 3,400 ms, as in the main SRC task. Videos for each speech sound were played six times, rendering 24 trials total. Altogether, the experiment lasted approximately 40 min.

#### Data processing and analysis

Audio recordings in the main SRC task were set to start at distractor video onset for 3,400 ms. RTs from recording onsets were measured manually on Praat. To obtain the true RTs from prompt onsets, RTs from recording onsets were corrected for video-onset latencies and SOA (see Wilt et al., 2022). Video onset latencies were obtained by averaging the difference between the expected audio onset in the SRC video stimuli and the observed audio onset in the video-onset detection task, for each participant and speech sound. Video onset latencies averaged 107 ms across participants (*SD* = 56 ms, *range* = -58 – 300 ms). We also computed SDs of the video-onset latencies for each combination of participant and syllable as an indicator of the variability in video-onset latencies per condition. Participants with SDs greater than 30 ms for one or more sounds were excluded from the experiment (four participants). The SDs for the remaining participants averaged 11 ms. To obtain our final RTs, RTs measured manually from recording onsets in the SRC task were hence corrected for SOA from video onset (1,000 ms, 1,200 ms, 1,400 ms) and for mean video-onset latency specific to the participant and syllable (*RT from prompt onset* = *RT from recording onset* – *SOA* – *mean* *video-onset* *latency*).

The full dataset comprised 11,214 observations. Thirty-eight trials (0.34%) were considered defective and removed: 21 recordings malfunctioned; one recording was obstructed by background noise; 12 recordings contained yawning or coughing; four recordings picked up a mobile phone notification and were excluded for risk of participant distractedness. A further 532 erroneous trials were removed (4.76%): 420 productions of the wrong or of multiple prompts; 83 missing answers; 29 anticipatory responses. 804 observations with RTs outside of three MADs from a participants’ mean in each experimental condition for correct trials were further excluded from the analyses. ERs averaged 4.26% (*SD* = 6.08%) for the native task, and 5.28% (*SD* = 7.56%) for the non-native task. The remaining 9,840 trials were included in the RT analyses.

The generalised mixed modelling procedure was identical to that in Experiment 1. The maximal random effect structure to converge comprised of by-participant intercepts and slopes for Compatibility, Nativeness and SOA.

### Results

**Fig. 4:**
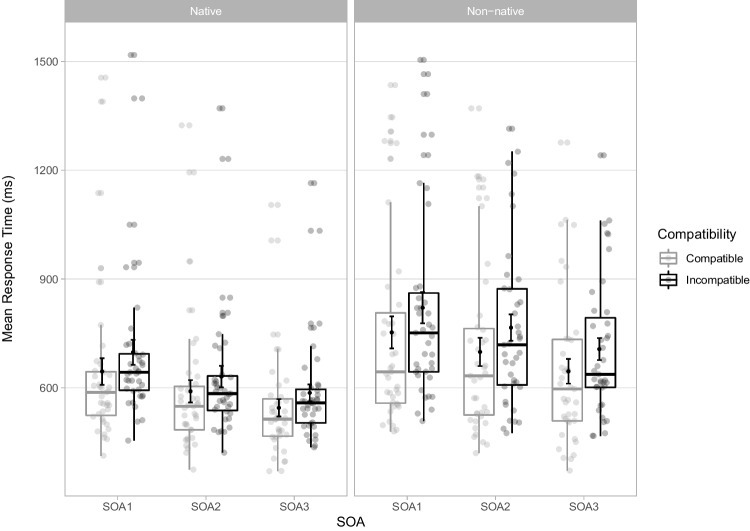
Mean response times (RTs) in milliseconds (ms) for correct trials in the stimulus-response compatibility (SRC) tasks for each experimental condition in Experiment 2. Points in the background show the raw mean RTs for each participant (points are offset on the x-axis for clarity). The boxplots indicate the first, second (median) and third quartiles, and whiskers indicate 1.5 times the interquartile range of the distribution. Black points in the foreground show the mean and error bars indicate standard errors

In all, 9,840 trials were analysed. Mean RTs for each experimental condition are displayed in Fig. 4 and OSM Appendix D, and the backward selection process is detailed in OSM Appendix E. The final model included main effects of Compatibility, Nativeness, SOA and the interaction Compatibility x Nativeness (Table 3).

**Table 3:** Final model of raw reaction times (RTs) in milliseconds (ms) using a gamma distribution and identity link function for Experiment 2

Fixed effect	Estimate	*SE*	t-value	p-value
(Intercept)	**728**	**3**	**221**	**< 2 x 10**^-16^***
Nativeness	**133**	**8**	**16**	**< 2 x 10**^-16^***
Compatibility	**50**	**5**	**11**	**< 2 x 10**^-16^***
SOA2-1	**-61**	**4**	**-17**	**< 2 x 10**^-16^***
SOA3-2	**-56**	**3**	**-17**	**< 2 x 10**^-16^***
Compatibility x Nativeness	**22**	**3**	**7**	**9.52 x 10**^-13^***

*Note:* SOA = stimulus-onset asynchrony. Bold entries indicate significant effects. * *p* < .05, **p < .01, ***p < .001

The main effect of Compatibility was significant, with slower RTs in incompatible (*M* = 701 ms, *SD* = 221 ms) than compatible trials (*M* = 646 ms, *SD* = 229 ms), with a mean compatibility effect of 55 ms (*SD* = 61 ms). RTs were slower for non-native (*M* = 732 ms, *SD* = 242 ms) than native sounds (*M* = 616 ms, *SD* = 193 ms). RTs decreased with SOA, from SOA1 (*M* = 729 ms, *SD* = 255 ms) to SOA2 (*M* = 671 ms, *SD* = 222 ms) to SOA3 (*M* = 621 ms, *SD* = 181ms). The compatibility effect was modulated by Nativeness, with larger effects for non-native (*M* = 66 ms, *SD* = 70 ms) than for native sounds (*M* = 45 ms, *SD* = 49 ms).

Experiment 2 replicated the main finding of Experiment 1: automatic imitation is enhanced in an SRC task with non-native speech sounds. However, in both experiments, participants produced native responses only when perceiving native distractors, and non-native responses only while perceiving non-native distractors. Hence, these results cannot rule out the possibility that the larger compatibility effects for non-native sounds reflect effects of *producing* unfamiliar speech actions rather than of *perceiving* them. Experiment 3 accounted for this confound by intermixing native and non-native responses and distractors within a single SRC task, allowing to disentangle perception-driven from production-driven effects.

## Experiment 3

### Methods

#### Participants

To obtain our sample of 40 participants per our pre-registration (AsPredicted #112675), 42 participants were recruited. All self-reported being native British-English speakers with normal hearing, normal or corrected-to-normal vision and no speech disorders or neurological disorders. The full list of languages spoken by participants is displayed in OSM Appendix F. Participants received £9 compensation or course credit for this one-hour experiment. Two participants were excluded for having error rates > 50% for one or more experimental condition. The final sample comprised of 40 participants (33 female, *M*_age_ = 23.05, *SD*_age_ = 5.11, range: 18–39 years).

#### Stimuli

**Fig. 5:**
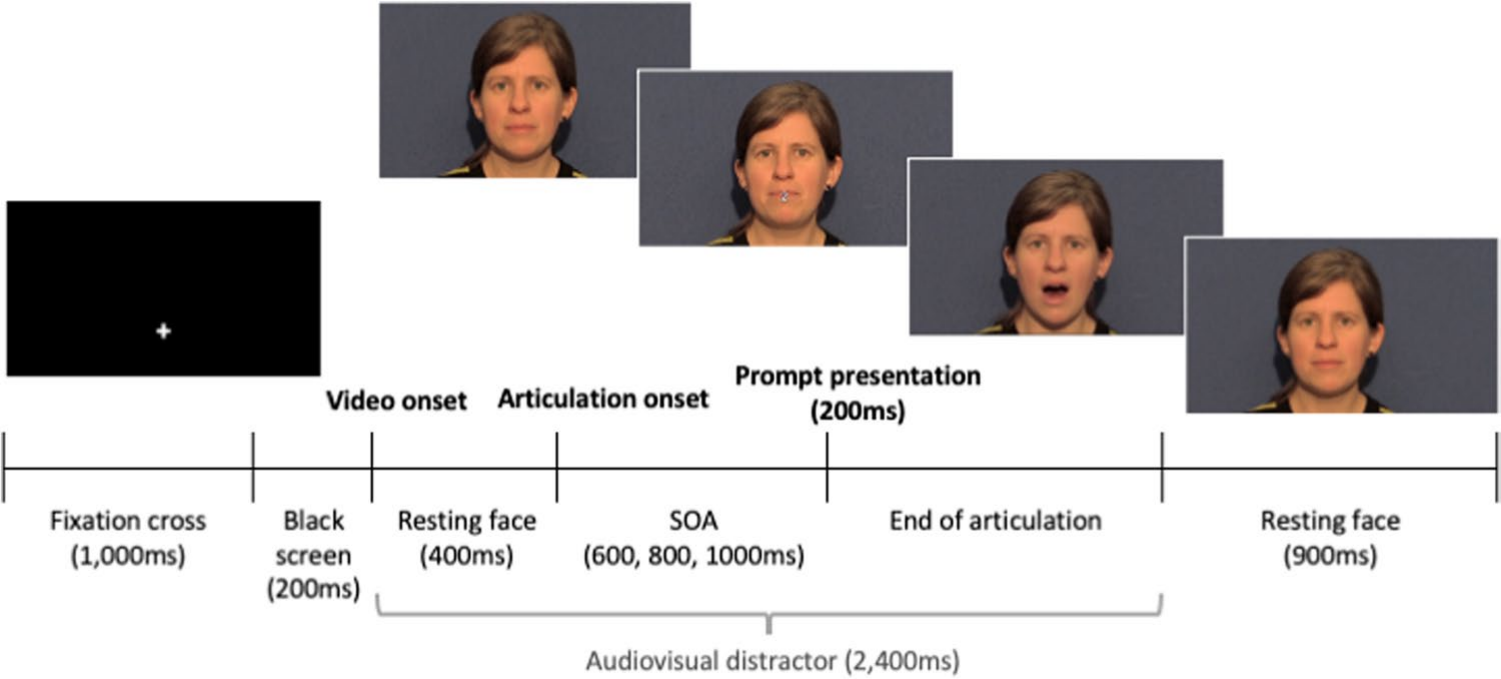
Stimulus-response compatibility (SRC) trial timeline for Experiment 3. Timings in parentheses represent durations

The video and audio recordings were the same as that used in Experiment 1 and Experiment 2. Videos and response prompts (Helvetica white font size 78 pt, black outline width 3.0) were aligned on Apple Final Cut Pro, and audio stimuli were aligned directly on Psychopy (Peirce et al., 2022). In contrast with the previous experiments, numerical prompts 1, 2, 3, 4 were used instead of symbols. This methodological choice was adopted in order to facilitate retention of the prompt-response pairings, as all four responses prompts were presented within blocks. As in the previous experiments, the response prompts appeared over the speaker’s mouth at one of three SOAs (600, 800 or 1,000 ms post articulation onset). Distractor videos were preceded by a white fixation cross at the location of the response prompt for 1,000 ms over a black screen, which then disappeared leaving the screen black for 200 ms before the onset of the distractor stimulus (cf. Fig. 5).

Videos were also created on Final Cut Pro for the first set of practice trials, in which participants viewed and responded to the numerical prompts with no distractors. These videos were identical to that used for the SRC trials except that the background distractor video was replaced by a black background, and prompts consistently appeared at 1,000 ms from video onset (equivalent to SOA1).

#### Procedure

The experiment was conducted in a soundproofed, light-controlled booth. Participants wore a Beyerdynamic DT 297 PV MK II headset as they completed the SRC task in Psychopy on a Dell PC. Participants first received brief oral instructions for the SRC task, before viewing more specific instructions displayed in Microsoft Powerpoint slides. In these slides, four videos were first displayed on the screen simultaneously, each preceded by the instructions “If you see ‘[number prompt]’, produce:”. Eight possible prompt-response pairings were counterbalanced across participants. The researcher played each video one after the other, from the sound paired with the symbol 1 to that paired with the symbol 4 in numerical order. Participants were asked to attempt to produce each sound directly after watching each video. The researcher then played the production instruction videos. Each of these videos was displayed on a single slide, with the paired response prompt displayed beside it. Once again, the videos were presented to the participants from prompts 1–4. Participants could view each production instruction video as many times as they wanted and produced each sound at least five times. Once they were comfortable producing each sound, participants completed the practice trials on Psychopy.

First, participant viewed a reminder of the SRC task instructions, as well as a reminder of the prompt-response pairings in which each of the number prompts 1–4 (Arial bold, white letter height 0.08) were presented one after the other over a black background for three seconds each while the associated sound played. Participants could view this reminder as many times as they wanted. Participants then completed a block of 12 practice trials in which no video or audio was included, but only number prompts over a black background appearing in pseudorandom order. That is, participants viewed the numerical prompts only (no distractors) and had to produce the associated speech sound as quickly as possible. Timings were set to mimic the SRC trial. This set of trials was included to ensure retention of the prompt-response pairings. Participants could choose to repeat this block as many times as possible until they felt comfortable with remembering the prompt-response pairings. The maximal number of blocks completed by a participant was three. Once they were ready, participants competed a single practice block of 36 practice trials (4 responses x 3 distractor conditions x 3 SOAs) of the SRC task (with video and audio stimuli), followed by 15 blocks of 36 trials in the main task (540 trials total). Altogether, the testing session lasted approximately 50 min.

Unlike in Experiments 1 and 2, participants produced and perceived all four sounds ([bɑ], [lɑ], [ʙɑ], [ɮɑ]) within a single SRC task. Crucially, we chose to pair each response with only three possible distractors. Hence, in each trial responses and distractors could either be compatible, incompatible with a native distractor, or incompatible with a non-native distractor (cf. Table 4). An incompatible distractor always differed from the target response in its place of articulation. This design choice was implemented as we were not interested in comparing incompatible response-distractor pairs that shared the place of articulation (i.e., [bɑ]-[ʙɑ] or [lɑ]-[ɮɑ]) as this would add a new level of complexity to the experiment and hinder the interpretability of results.

**Table 4:** Response–distractor syllable pairings in Experiment 3 and their associated experimental condition

Response condition	Response syllable	Distractor condition	Distractor syllable
Native	[bɑ]	Compatible	[bɑ]
		Incompatible – native	[lɑ]
		Incompatible – non-native	[ɮɑ]
	[lɑ]	Compatible	[lɑ]
		Incompatible – native	[bɑ]
		Incompatible – non-native	[ʙɑ]
Non-native	[ʙɑ]	Compatible	[ʙɑ]
		Incompatible – native	[lɑ]
		Incompatible – non-native	[ɮɑ]
	[ɮɑ]	Compatible	[ɮɑ]
		Incompatible – native	[bɑ]
		Incompatible – non-native	[ʙɑ]

#### Data processing and analysis

Participants’ vocal responses were recorded using a Beyerdynamic DT 297 PV MK II headset microphone. Recordings started at video onset for 3,300ms. Response annotations and RT measurements were manually determined on Praat.

The full dataset for 40 participants comprised 21,504 observations. For the RT analyses, 2,382 erroneous trials were removed (11.08%): 1,329 wrong answers; 994 missing answers; 59 anticipatory responses (RT < 200 ms). ERs averaged 9.95% (*SD* = 12.98%) for native responses and 12.26% (*SD* = 13.69) for non-native responses. A further 1,466 observations were removed with RTs over three MADs from each participant’s mean in each experimental condition. The remaining 17,660 trials were included in the analyses.

Raw RTs for correct trials were analysed with general linear mixed effects models using the *lme4* package in R (Bates et al., 2014). Fixed factors were Response (native vs. non-native), Distractor (compatible vs. incompatible–native vs. incompatible–non-native), SOA (SOA1 (600 ms), SOA2 (800 ms), SOA3 [1,000 ms)) and their interactions. Response was coded as -0.5 and 0.5 for the native and non-native conditions, respectively, and backward difference coding was used for SOA as in Experiments 1 and 2. Backward difference coding was also used for Distractor to allow for the sequential comparison of incompatible–native versus compatible trials (level 2-1) and of incompatible–non-native trials versus incompatible–native trials (level 3-2). To demonstrate enhanced automatic imitation for non-native distractors, RTs should be larger for incompatible native than for compatible trials (significant Distractor level 2-1) and larger for incompatible–non-native than for incompatible–native trials (significant Distractor level 3-2).

We assumed a gamma distribution and identity link function. The maximal random effect structure comprised of by-participant random intercepts and slopes for Response. The backward model selection process was identical to that adopted in Experiments 1 and 2.

### Results

**Fig. 6:**
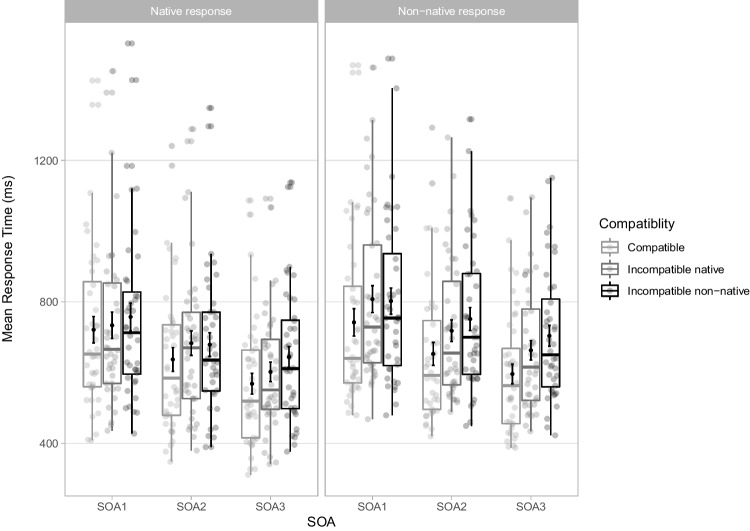
Mean response times (RTs) in milliseconds (ms) for correct trials in the stimulus-response compatibility (SRC) tasks for each experimental condition in Experiment 3. Points in the background show the raw mean RTs for each participant (points are offset on the x-axis for clarity). The boxplots indicate the first, second (median) and third quartiles, and whiskers indicate 1.5 times the interquartile range of the distribution. Black points in the foreground show the mean and error bars indicate standard errors

In all, 17,660 trials were analysed. Mean RTs for each experimental condition are displayed in Fig. 6 and OSM Appendix G, and the backward selection procedure is detailed in OSM Appendix H. When running exploratory analyses controlling for effects of Prompt (1-4) on RTs and Compatibility, Prompt significantly improved model fit and hence was included in the model (cf. OSM Appendix G). There was no evidence for the inclusion of the interaction Prompt x Compatibility, hence Prompt did not modulate compatibility effects. The final model included main effects Response, Distractor, SOA and Prompt, as well as the interactions Response x Distractor and Distractor x SOA (Table 5).

**Table 5:** Final model of raw reaction times (RTs) in milliseconds (ms) using a gamma distribution and identity link function for Experiment 3

Fixed effect	Estimate	*SE*	t-value	*p*-value
(Intercept)	**678**	**2**	**277.138**	**< 2 x 10**^-16^***
Response	**45**	**2**	**21.267**	**< 2 x 10**^-16^***
Distractor2-1	**49**	**2**	**24.958**	**< 2 x 10**^-16^***
Distractor3-2	**19**	**2**	**8.981**	**< 2 x 10**^-16^***
SOA2-1	**-62**	**2**	**-30.371**	**< 2 x 10**^-16^***
SOA3-2	**-47**	**1**	**-32.745**	**< 2 x 10**^-16^***
Prompt	**16**	**1**	**11.925**	**< 2 x 10**^-16^***
Response x Distractor2-1	**33**	**2**	**14.338**	**< 2 x 10**^-16^***
Response x Distractor3-2	2	2	0.858	0.391
Distractor2-1 x SOA2-1	**16**	**2**	**7.313**	**2.61 x 10**^-13^***
Distractor3-2 x SOA2-1	2	2	1.175	0.240
Distractor2-1 x SOA3-2	-3	3	-1.053	0.292
Distractor3-2 x SOA3-2	**23**	**2**	**10.589**	**< 2 x 10**^-16^***

*Note:* SOA = stimulus-onset asynchrony. Bold entries indicate significant effects. * *p* < .05, ** *p* < .01, *** *p* < .001

There main effect of Response was significant, with slower RTs for non-native (*M* = 716 ms, *SD* = 216 ms) than native responses (*M* = 670 ms, *SD* = 219 ms). The main effect of SOA was significant, with RTs decreasing from SOA1 (*M* = 761 ms, *SD* = 240 ms) to SOA2 (*M* = 687 ms, *SD* = 210 ms) to SOA3 (*M* = 630, *SD* = 184). The main effect of Distractor was significant, with participants responding faster in compatible (*M* = 653 ms, *SD* = 218 ms) than incompatible–native trials (*M* = 701 ms, *SD* = 216 ms), and for incompatible–native than incompatible–non-native trials (*M* = 723 ms, *SD* = 218 ms). Automatic imitation effects can be estimated by subtracting the mean RTs in the compatible condition from the mean RT of the incompatible–native or the incompatible–non-native distractor conditions. The automatic imitation effect averaged 48 ms (*SD* = 76 ms) for the incompatible–native distractors, and 70 ms (*SD* = 70 ms) for incompatible–non-native distractors. The effect of Distractor was further modulated by Response. The RT difference between incompatible–native and compatible distractor trials (Distractor2-1) increased significantly for non-native responses, while the RT difference between incompatible–native and incompatible–non-native distractor trials (Distractor3-2) did not. That is, producing non-native responses increased automatic imitation of native distractors (native response: *M* = 31 ms, *SD* = 69 ms; non-native response: *M* = 66 ms *SD* = 79 ms) and non-native distractors (native response: *M* = 51 ms, *SD* = 61 ms; non-native response: *M* = 89 ms, *SD* = 74), yet the size of the effect was similar for native and non-native distractors (+35–38 ms). The effect of Distractor was also modulated by SOA. The RT difference between incompatible–native and compatible trials increased significantly from SOA1 to SOA2 (+17 ms) but did not change significantly from SOA2 to SOA3 (-6 ms), while the difference between incompatible–non-native and incompatible–native trials increased significantly from SOA2 to SOA3 (+28 ms) but not from SOA1 to SOA2 (+7 ms).

Results from Experiment 3 confirmed that perceiving non-native speech sounds enhanced automatic imitation relative to perceiving native speech sounds, supporting the view that covert imitation serves a compensatory role in speech perception (Wilson & Knoblich, 2005). The experiment further uncovered an effect of producing non-native speech actions on enhancing automatic imitation.

## General discussion

The present study aimed to establish automatic imitation effects for native and non-native speech sounds, to clarify the role of covert imitation in speech perception. A secondary goal was to validate the appropriateness of conducting speech SRC research online. In a laboratory-based study (Experiment 1) and its online replication (Experiment 2), participants completed two stimulus-response compatibility (SRC) tasks, one with native speech sounds and one with non-native speech sounds. These sounds were then intermixed in a single task in a final lab-based experiment (Experiment 3) to control for production-driven effects.

Experiment 1 uncovered an overall automatic imitation effect of 49ms. RTs were slower in the non-native task by 73 ms, reflecting the challenge elicited by producing unfamiliar actions. RTs decreased at longer SOAs, in line with previous speech SRC studies (Adank et al., 2018; Kerzel & Bekkering, 2000; Galantucci et al., 2009; Ghaffarvand Mokari et al., 2020; Wilt et al., 2022; Wu et al., 2019). Crucially, the automatic imitation effect was larger in the non-native task (63 ms) than in the native task (36 ms). The compatibility effect for native sounds conforms with effects reported in previous SRC speech studies using choice response paradigms with native consonants, ranging from 13–42 ms (Kerzel & Bekkering, 2000, Experiments 1 and 2; Galantucci et al., 2009, Experiments 1-2; Jarick & Jones, 2009; Wilt et al., 2022; Wu et al., 2019). Further, the difference in automatic imitation effects between native and non-native tasks was smaller at SOA1 (6 ms) compared to SOA2 (45 ms) and SOA3 (33 ms). The small difference in compatibility effects between Nativeness conditions at SOA1 likely reflects the fact that little articulatory information was present at that point in the distractor, hence the distinction between native and non-native sounds was not yet evident.

Experiment 2 replicated Experiment 1 online, resulting in an overall compatibility effect of 55 ms. RTs were slower in the non-native task by 116ms. As in Experiment 1 and consistent with previous work, RTs decreased with SOA. Crucially, automatic imitation effects were larger in the non-native (66 ms) than in the native task (45 ms). This replication of our main finding corroborates that speech SRC tasks can successfully be run online (Wilt et al., 2022) and demonstrates the robustness of the effect. Contrary to results of Experiment 1, however, the difference in automatic imitation effects between tasks was not modulated by SOA. In the online experiment, RT measurements were adjusted for latencies between recording and video stimuli onsets, which were derived from means rather than obtained on a trial-by-trial basis. Hence, RT measurements were less precise in Experiment 2 than in Experiment 1. The added noise likely reduced the power to detect subtle modulatory effects of SOA.

In Experiment 3, participants produced and perceived all four experimental sounds within the same task blocks. This design enabled to disentangle the effects of perceiving non-native speech sounds (main effect of Distractor) from effects driven by producing non-native speech sounds (Distractor x Response interaction), as both effects were confounded in Experiments 1 and 2. As in the previous experiments, RTs were slower when producing non-native responses by 46 ms, and decreased with SOA. Crucially, automatic imitation was greater for non-native distractors (70 ms) than for native distractors (48 ms). Further, compatibility effects were enhanced for non-native responses, and to a similar degree when perceiving incompatible–non-native distractors (+38 ms) or incompatible–native distractors (+35 ms). Analogously, the effect of perceiving incompatible–non-native distractors compared to incompatible–native distractors was similar when producing a native sound (+20 ms) than when producing a non-native sound (+23 ms).

Together, these experiments demonstrate that automatic imitation is enhanced when perceiving non-native sounds compared to native sounds. This finding was replicated in all three experiments, persisting online (Experiment 2) as well as when controlling for production-driven effects (Experiment 3). Our results support the prediction from Wilson and Knoblich that covert imitation preferentially supports speech perception under challenging listening conditions. To overcome the perceptual challenge associated with processing unfamiliar speech sounds, listeners may recruit motor processes more when perceiving non-native speech. In contrast, our results dispute the prediction that covert imitation is enhanced when perceiving familiar speech (Pickering & Garrod, 2013).

Several properties of speech actions may explain the apparent discrepancy between our findings and the literature on non-speech actions. Studies on the covert imitation of manual and bodily actions have typically reported enhanced automatic imitation for more familiar actions (Calvo-Merino et al., 2005; Cook et al., 2010; Gillmeister et al., 2008; Haslinger et al., 2005; Heyes et al., 2005; Margulis et al., 2009). Unlike speech actions, these actions typically do not represent meaningful categories acquired during development and hence their covert imitation may be more readily modulable by experience. This point could explain why automatic imitation of speech was not significantly reduced following counter-mirror training in Wu et al.’s (2019) study, while similar studies with manual actions consistently eliminated automatic imitation through counter-mirror training (Cook et al., 2010; Gillmeister et al., 2008; Heyes et al., 2005). Best’s Perceptual Assimilation Model (PAM, Best et al., 2009) could bridge the gap between our findings and the literature on manual actions, as well as action perception models such as the Theory of Even Coding (Hommel, 2009, 2019) and the Associative Sequence Learning model (Heyes, 2005, 2011b), which state that sensorimotor experience is necessary to establish action-perception links. The model posits that unfamiliar speech sounds are perceived as good or poor productions of the most articulatorily similar native phoneme through an assimilation process. From this perspective, participants may have utilised their sensorimotor experience with native speech to support the perception of novel speech sounds.

Another novel finding in the present research was the detection of production-driven effects on automatic imitation in Experiment 3, where automatic imitation was found to not only be enhanced by perceiving non-native speech distractors but also by producing non-native speech responses, relative to perceiving and producing native sounds. An effect of production on automatic imitation has previously been observed by Virhia et al. (2019). Participants completed speech SRC tasks in which audiovisual distractors could be neutral or emotional (happy/angry), and responses were produced in a neutral or emotional (happy/angry) manner. Automatic imitation was enhanced when producing emotional responses (41 ms, vs. 29 ms for neutral responses) but not when perceiving emotional distractors (35 ms, vs. 36 ms for neutral distractors), a result the authors interpreted as demonstrating an effect of emotional state on automatic imitation. Within the context of our present findings, Virhia et al.’s results could instead reflect an effect of production effort on automatic imitation.

The precise mechanisms underlying this effect, however, are not evident. It is possible that participants attended to the distractors more when producing non-native responses to monitor and correct their responses. However, if this were the case one would expect larger compatibility effects in the non-native task in Experiments 1 and 2 than for non-native response-distractor pairs in Experiment 3, where native and non-native distractors were presented within the same blocks and hence distractors were less likely to be relevant for non-native production correction. This was not the case, with automatic imitation effects in the non-native task averaging 63ms and 66ms in Experiments 1 and 2, respectively, compared to 89ms for the non-native response – non-native distractor condition in Experiment 3. Further, a self-monitoring explanation would predict a superadditive effect of producing and perceiving non-native speech sounds, where the effects of producing non-native sounds are enhanced when perceiving non-native sounds. However, Experiment 3 found that the effects of producing non-native responses on automatic imitation were similar when perceiving native and non-native distractors (+35–38 ms). Hence, a self-monitoring explanation seems unlikely.

Second, the challenge introduced by having to produce an unfamiliar speech action may have enhanced cognitive load, thereby diminishing available central processing resources (Lavie et al., 2004; Matthews, 2000; Plass et al., 2010), for example, inhibition to suppress the automatic imitative response tendency. RTs were greater when producing non-native responses across the three experiments, reflecting that participants found these more difficult to produce than native sounds. This view could also explain why compatibility effects for non-native response-distractor pairs were smaller in Experiments 1 and 2 than in Experiment 3, as in the latter experiment participants had to retain and produce four responses throughout the task, enhancing working memory load. The increased error rate in Experiment 3 (11.08%) compared to Experiments 1 (5.08%) and 2 (4.76%) likely reflects this additional challenge. Interestingly, however, previous studies have found no effect of cognitive load on automatic imitation of manual actions (Ramsey et al., 2019; Trotter et al., 2023), or even decreased compatibility effects with increasing cognitive load (van Leeuwen et al., 2009). A TMS study by Puglisi et al. (2018) found that increased cognitive load eliminated imitative motor activation during observation of manual actions. Hence, it is unclear whether our finding of a facilitatory effect of production effort on automatic imitation can be interpreted as reflecting effects of cognitive load. Further work is necessary to confidently identify the mechanisms underlying the effects of producing unfamiliar speech actions in enhancing compatibility effects.

## Conclusion

In three experiments, we showed that automatic imitation is enhanced when perceiving non-native speech sounds compared to native sounds. This finding held in an online setting as well as when controlling for production-driven effects. The results support a compensatory function of covert imitation in speech perception (Wilson & Knoblich, 2005) and challenge the integrated theory of language (Pickering & Garrod, 2013). Our final experiment additionally uncovered a significant effect of non-native speech production in facilitating automatic imitation.

### Supplementary Information

Below is the link to the electronic supplementary material.

Supplementary file1 (DOCX 52 KB)

Supplementary file2 (DOCX 39 KB)
