# Supplementary material for: Automatic imitation of speech is enhanced for non-native sounds
Source: Psychon Bull Rev. 2023 Oct 17;31(3):1114–30. doi: 10.3758/s13423-023-02394-z (PMC11192695; doi:10.3758/s13423-023-02394-z)
Supplement: Supplementary file 2 — Supplementary file2 (DOCX 39 KB) [file 13423_2023_2394_MOESM2_ESM.docx]

APPENDIX

Appendix A: List of languages spoken by participants included in Experiment 1.

| Language | N participants |
| --- | --- |
| Arabic | 2 |
| Bengali | 1 |
| Farsi | 1 |
| French | 1 |
| Greek | 1 |
| Italian | 1 |
| Japanese | 1 |
| Persian | 1 |
| Polish | 1 |

Appendix B: Key articulatory event timings of the audiovisual speech stimuli.

**Table B1.** Visual articulation and audio onset and durations in milliseconds (ms) for each distractor video.

| Distractor | Visual articulation onset | Audio onset | Auditory stimuli duration | | |
| --- | --- | --- | --- | --- | --- |
|  |  |  | Consonant | Vowel | Total |
| [ba] | 400 | 1520 | 6 | 681 | 687 |
| [la] | 400 | 1178 | 262 | 544 | 806 |
| [ʙɑ] | 400 | 1470 | 132 | 629 | 761 |
| [ɮɑ] | 400 | 1135 | 433 | 602 | 1035 |
| *Note: [*ba] and [la] are the native sounds, [ʙɑ] and [ɮɑ] are the non-native sounds. | | | | |  |

Appendix C: Mean RTs and SDs for each experimental condition in Experiment 1.

**Table C1.** Mean RTs and SDs for each experimental condition in Experiment 1.

| Nativeness | SOA | Compatibility | RT (ms) | *SD* (ms) |
| --- | --- | --- | --- | --- |
| Native | SOA1 | Compatible | 686 | 193 |
|  |  | Incompatible | 731 | 184 |
|  | SOA2 | Compatible | 646 | 177 |
|  |  | Incompatible | 673 | 160 |
|  | SOA3 | Compatible | 594 | 137 |
|  |  | Incompatible | 629 | 126 |
| Non-native | SOA1 | Compatible | 756 | 205 |
|  |  | Incompatible | 807 | 187 |
|  | SOA2 | Compatible | 702 | 183 |
|  |  | Incompatible | 773 | 163 |
|  | SOA3 | Compatible | 646 | 145 |
|  |  | Incompatible | 714 | 130 |
| *Note:* ms = milliseconds*.* | | | | |

Appendix D: Mean RTs and SDs for each experimental condition in Experiment 2.

**Table D1.** Mean RTs and SDs for each experimental condition in Experiment 2.

| Nativeness | SOA | Compatibility | RT (ms) | *SD* (ms) |
| --- | --- | --- | --- | --- |
| Native | SOA1 | Compatible | 645 | 229 |
|  |  | Incompatible | 698 | 214 |
|  | SOA2 | Compatible | 590 | 193 |
|  |  | Incompatible | 631 | 186 |
|  | SOA3 | Compatible | 545 | 149 |
|  |  | Incompatible | 586 | 145 |
| Non-native | SOA1 | Compatible | 753 | 276 |
|  |  | Incompatible | 821 | 269 |
|  | SOA2 | Compatible | 699 | 244 |
|  |  | Incompatible | 766 | 227 |
|  | SOA3 | Compatible | 645 | 213 |
|  |  | Incompatible | 707 | 190 |

*Note:* ms = milliseconds*.*

Appendix E: Backward model selection procedure and saturated model for Experiment 2.

The maximal random effect structure to converge and pass singularity checks comprised of by-participant random intercepts and slopes for Compatibility, Nativeness and SOA. Starting with the saturated model (Table E1), we performed backward selection to determine the best fitting model. The three-way interaction Compatibility x Nativeness x SOA did not improve model fit and was removed from the model (χ^2^(2)= 1.758, *p*=.415). The two-way interaction Compatibility x SOA was removed next (χ^2^(2)=2.605, *p*=.272), followed by Nativeness x SOA (χ^2^(2)=4.081, *p*=.130). Crucially, Compatibility x Nativeness was found to significantly improve model fit and was kept in the model (χ^2^(1)=21.358, *p*=3.81 x 10^-6^). SOA significantly improved model fit and was kept in the model (χ^2^(2)=28.344, *p*=7.001 x 10^-7^). No other factors were removed as they were included in higher order effects. The final model included the main effects of Compatibility, Nativeness, SOA and the interaction Nativeness x Compatibility.

**Table E1.** Saturated model of raw reaction times (RTs) in milliseconds (ms) using a gamma distribution and identity link function for Experiment 2.

| Fixed Effect | Estimate | *SE* | t-value | p-value |
| --- | --- | --- | --- | --- |
| **(Intercept)** | **728** | **6** | **123.83°** | **< 2 x 10^-16^***** |
| **Nativeness** | **133** | **5** | **27.494** | **< 2 x 10^-16^***** |
| **Compatibility** | **51** | **4** | **14.170** | **< 2 x 10^-16^***** |
| **SOA2-1** | **-61** | **4** | **-16.817** | **< 2 x 10^-16^***** |
| **SOA3-2** | **-58** | **3** | **-18.553** | **< 2 x 10^-16^***** |
| **Nativeness x Compatibility** | **22** | **3** | **6.941** | **3.89 x 10^-12^***** |
| **Nativeness x SOA2-1** | **10** | **3** | **3.456** | **0.0005**** |
| **Nativeness x SOA3-2** | **-11** | **3** | **-3.565** | **0.0003**** |
| Compatibility x SOA2-1 | -7 | 4 | -1.928 | 0.054 |
| Compatibility x SOA3-2 | -1 | 3 | -0.291 | 0.771 |
| **Nativeness x Compatibility x SOA2-1** | **15** | **3** | **4.598** | **4.27 x 10^-6^***** |
| Nativeness x Compatibility x SOA3-2 | -4 | 4 | -1.001 | 0.317 |

| *Note:* SOA = Stimulus-Onset Asynchrony. * *p*<.05, ***p*<.01, ***p<.001. |
| --- |

Appendix F: List of languages spoken by participants included in Experiment 3.

| Language | N participants |
| --- | --- |
| Arabic | 2 |
| Bengali | 4 |
| French | 2 |
| Greek | 1 |
| Hindi | 2 |
| Irish | 1 |
| Korean | 1 |
| Mandarin | 1 |
| Marwari | 1 |
| Nigerian | 1 |
| Punjabi | 1 |
| Portuguese | 2 |
| Spanish | 4 |
| Swahili | 1 |
| Urdu | 3 |

Appendix G: Mean RTs and SDs for each experimental condition in Experiment 3.

**Table G1.** Mean RTs and SDs for each experimental condition in Experiment 3.

| Response | SOA | Distractor | RT (ms) | *SD* (ms) |
| --- | --- | --- | --- | --- |
| Native | SOA1 | Compatible | 721 | 236 |
|  |  | Incompatible native | 734 | 237 |
|  |  | Incompatible non-native | 757 | 248 |
|  | SOA2 | Compatible | 637 | 213 |
|  |  | Incompatible native | 683 | 219 |
|  |  | Incompatible non-native | 679 | 212 |
|  | SOA3 | Compatible | 568 | 185 |
|  |  | Incompatible native | 602 | 175 |
|  |  | Incompatible non-native | 644 | 188 |
| Non-native | SOA1 | Compatible | 742 | 244 |
|  |  | Incompatible native | 808 | 241 |
|  |  | Incompatible non-native | 802 | 234 |
|  | SOA2 | Compatible | 653 | 205 |
|  |  | Incompatible native | 718 | 194 |
|  |  | Incompatible non-native | 751 | 207 |
|  | SOA3 | Compatible | 596 | 174 |
|  |  | Incompatible native | 663 | 173 |
|  |  | Incompatible non-native | 704 | 186 |

*Note:* ms = milliseconds*.*

Appendix H: Backward model selection procedure and saturated model for Experiment 3.

The maximal random effect structure to converge and pass singularity checks included by-participant intercepts and slopes for Response. Starting with the saturated model (Table H1), we performed backward selection to determine the best fitting model. The three-way interaction Response x Congruency x SOA improved model fit (χ^2^(4)=9.755, *p*=0.045, BF_10_= 3.059 x 10^-7^). As the p-value was barely significant and the BF_10_ very decisively in favour of the simple model, we chose to stray from our pre-registration and remove the three-way interaction from the model. Next, the two-way interaction Response x SOA was removed as it did not improve model fit (χ^2^(2)=0.923, *p*=0.630). Response x Congruency improved model fit and was kept in the model (χ^2^(2)=44.851, *p*=1.823 x 10^-10^), as was Congruency x SOA (χ^2^(4)=13.096, *p*=2.927 x 10^-6^). No main effects were removed as they were included in significant two-way interactions. In order to assess our decision to remove the three-way interaction Response x Congruency x SOA from the model, we also ran a chi-squared test comparing our final model with the saturated model. The saturated model was not a better fit than the simpler model (χ^2^(6)=10.678, *p*=0.099), hence the three-way interaction did not improve model fit and the simpler model was retained. Finally, we ran exploratory analyses to check whether Prompt (1-4) had an effect on RTs and, more importantly, on compatibility effects. We chose to run this analysis as participants learned the prompt-response pairings in a specific order (1-4) and hence it is possible that they responded more quickly to 1 than to 4, for e.g.. We tested for the addition of Prompt (1-4) and Prompt x Compatibility to our model. The inclusion of Prompt benefitted model fit (χ^2^(1)=96.638, *p*<2.2 x 10^-16^), with RTs increasing with higher numerical prompts. Crucially, the interaction Prompt x Compatibility did not improve model fit (χ^2^(2)=4.37, *p*=0.113). Our final model hence included main effects of Response, Distractor, SOA, Prompt as well as the interactions Response x Distractor and Distractor x SOA.

**Table G1**. Saturated model of reaction times (RTs) in milliseconds (ms) using a gamma distribution and inverse link function for Experiment 3.

| Fixed Effect | Estimate | *SE* | t-value | p-value |
| --- | --- | --- | --- | --- |
| **(Intercept)** | **718** | **5** | **134.483** | **< 2 x 10^-16^***** |
| **Response** | **45** | **2** | **19.259** | **< 2 x 10^-16^***** |
| **Distractor2-1** | **49** | **2** | **27.462** | **< 2 x 10^-16^***** |
| **Distractor3-2** | **19** | **2** | **8.007** | **1.18 x 10^-16^***** |
| **SOA2-1** | **-62** | **3** | **-21.698** | **< 2 x 10^-16^***** |
| **SOA3-2** | **-47** | **2** | **-19.913** | **< 2 x 10^-16^***** |
| **Response:Distractor2-1** | **34** | **3** | **11.427** | **< 2 x 10^-16^***** |
| Response:Distractor3-2 | 1 | 4 | 0.176 | 0.860 |
| Response:SOA2-1 | 2 | 2 | 0.725 | 0.462 |
| Response:SOA3-2 | 3 | 2 | 1.484 | 0.138 |
| **Distractor2-1:SOA2-1** | **15** | **3** | **4.886** | **1.03 x 10^-6^***** |
| Distractor3-2:SOA2-1 | 5 | 3 | 1.839 | 0.066 |
| Distractor2-1:SOA3-2 | -2 | 3 | -0.700 | 0.484 |
| **Distractor3-2-1:SOA3-2** | **22** | **2** | **9.534** | **< 2 x 10^-16^***** |
| **Response:Distractor2-1:SOA2-1** | **-25** | **5** | **-5.225** | **1.74 x 10^-7^***** |
| **Response:Distractor3-2:SOA2-1** | **48** | **3** | **17.744** | **< 2 x 10^-16^***** |
| **Response:Distractor2-1:SOA3-2** | **7** | **3** | **2.491** | **0.013*** |
| **Response:Distractor3-2:SOA3-2** | **-31** | **3** | **-10.889** | **< 2 x 10^-16^***** |
| *Note:* SOA= Stimulus-Onset Asynchrony. * *p*<.05, ***p*<.01, ***p<.001 | | | | |
