# Supplementary material for: Automatic imitation of speech is enhanced for non-native sounds
Source: Psychon Bull Rev. 2023 Oct 17;31(3):1114–30. doi: 10.3758/s13423-023-02394-z (PMC11192695; doi:10.3758/s13423-023-02394-z)
Supplement: Supplementary file 1 — Supplementary file1 (DOCX 52 KB) [file 13423_2023_2394_MOESM1_ESM.docx]

London, July 2023

Dear Prof. Meyers

We would like to thank you and the reviewers for your comments and suggestions, and we appreciate the chance to resubmit. The suggestions regarding our statistical analyses were particularly helpful and informative. While running the analyses as suggested by reviewer 2 and reviewer 3, we uncovered an error in our original dataset for Experiment 1. After correction of this mistake, error rates were slightly altered and hence we had to exclude one further participant (for having an overall ER >3SD from the group mean). The analysis for the corrected dataset with 49 participants is now reported in this revision. Note that our results and conclusions remain the same. We discuss in the rebuttal below how we have addressed all comments in the revised paper. Primarily, since submitting the original manuscript, we have run and analysed a third experiment addressing the confound in the original experiments. This significant addition has required a major re-write and we have chosen to submit a revised manuscript without marking the changes made. If required, we can submit a manuscript with changes marked, but note that this would entail underlining a majority of the manuscript. Thank you for considering our paper for publication in Psychonomic Bulletin & Review.

Yours sincerely,

Hannah Wilt (corresponding author)

Yuchunzi Wu

Bronwen G. Evans

Patti Adank

*Reviewer: 1

Comments to the Author*

*This is a remarkably well-written and clear manuscript showing an enhanced SRC effect when participants hear and produce non-native syllables vs. native syllables. One particularly strong point is that most critical findings were replicated in an online environment, which suggests the effect is robust to noise. I very much appreciated how the authors carefully documented their procedure, exclusion criteria and analysis choices. I have however some relatively minor suggestions for improvement that I would recommend are addressed before acceptance.

1. The main suggestion is that the production-based explanation for the findings should be mentioned much earlier in the manuscript, ideally in the introduction. I think it would benefit the manuscript in terms of transparency if the authors were upfront about the fact that they cannot rule put this alternative explanation for their findings. I suspect (?) they were going to address it through the training aspect of the study which was cut short by COVID. If that's right, then even more reason to be upfront about it as it is not the authors' fault that they could not follow through with their plans (and these resullts are still worth publishing!).*

Response: We now mention the production confound in the Introduction, and report results of a third experiment which accounted for this confound. Lines 150-153: *“In Experiment 3, participants produced and perceived native and non-native sounds within a single SRC task, allowing to disentangle effects of perceiving non-native distractors from potential effects of producing unfamiliar speech actions on automatic imitation.”*

*2. Please report full information on additional languages the participants were exposed to/knew (if available). While only one of the participants knew Welsh, and was rightly excluded from the sample, given all participants were native speakers of British English it is conceivable they may have had at least some degree of exposure to Welsh sounds (through news, place names etc.). Could you comment briefly on this?*

Response: We have now added a full report of the additional languages spoken by participants in Experiments 1 and 3 in Appendix A and Appendix F respectively (participants in online Experiment 2 were all English monolinguals). When selecting the non-native sounds for our experiments, several factors had to be considered. E.g., the sounds had to be producible by native English speakers, and distinguishable from one another both visually and auditorily. We also wanted each non-native sound to share its place of articulation with a native sound, to minimise the articulatory distance between incompatible pairs in the SRC tasks. While it is possible that some participants had a minimal degree of exposure to [ɮ] through Welsh names etc., such exposure would be very limited, particularly in comparison to experience producing and perceiving the native sounds. The question of minor exposure to non-native sounds would have been an issue regardless of the sounds selected and can unfortunately not be well controlled for.

*3. It would help to define criteria for participant exclusion based on error rates more clearly (e.g., state how an error was defined before you say some participants nade 50% errors). Also it seems important to report error rates separately by condition (at the very least native vs. non-native) as it seems likely participants would have produced the wrong sound more often when it was non-native?*

Response: We now specify how errors were defined (lines 236-241: *“Errors were defined as productions of the wrong or of multiple responses, missing answers, or anticipatory responses with RTs<200ms. For the non-native sounds, productions were considered erroneous if they could not be clearly auditorily identified as attempts to produce either [ʙɑ] or [ɮɑ], and if the spectrogram did not show clear turbulence (for [ʙɑ] and [ɮɑ]) and/or at least one vocal tract resonance portion (for [ʙɑ] only).”),* and report error rates separately for native and non-native responses in each experiment (lines 244-245, lines 428-429, lines 556-557).

*4. How were the factor coded in the lmer models? Were they dummy coded? If so, this has implications for the interpretation of main effects and interactions*

Response: Following comments by Reviewer 2 and Reviewer 3, we have reanalysed our data using centered contrasts and backward difference coding. The coding scheme is described in the Methods section, lines 249-258.

*5. Figure 2 and 4 - please make sure the figures display conditions in the same order, for maximum comparability. Also, the captions refer to individual participants' means being displayed but they did not appear in my version of the proof at least*

Response: Figures 2 and 4 now display conditions in the same order. While individual means appeared in our version of the original manuscript, they were not visible in the online version. We have now uploaded the figures as separate files as well and hope that these are now visible.

*6. Please clarify how the size of the overall compatibility effect was derived and how it maps onto the estimates from the lmer model.*

Response: The compatibility effect is computed by subtracting RTs for compatible trials from RTs for incompatible trials, as described in the Introduction lines 80-81. Mean RTs are first aggregated across participant and condition, which is now specified in lines 300-301: *“computed from aggregated RTs per participant and experimental condition”*. As we now use contrast coding, the effect maps onto the Compatibility factor estimate. Any minor differences in reported automatic imitation effects and estimates (e.g., 55ms and 50ms respectively in Experiment 2) are due to the statistical models capturing additional complexities in the data, e.g., variability of the random effects and interactions between fixed factors.

*7. Please provide the OSF link*

Response: The OSF link has been added in the manuscript, line 760 (<https://osf.io/zbqng>).

Reviewer: 2

Comments to the Author

*This is a very interesting study which adds to what is known about how and why compatibility effects occur in stimulus-response compatibility (SRC) tasks with spoken distractors. The main finding is that a lack of familiarity (or nativeness) with the responses/distractors gives rise to a larger interference effect, suggesting that covert imitation of speech may be exploited to a greater extent in these more challenging circumstances. The study is introduced clearly and concisely, and a range of relevant literature is cited. Furthermore, the descriptions of the two experiments and results are generally clear. Finally, the discussion and conclusions are consistent with the findings. Overall, this submission is well suited for a Brief Report.*

*Abstract
This is OK. Here the authors describe what is meant by an “automatic imitation effect” and that this “indexes the degree to which the distractor stimulus is covertly imitated”. While I understand the authors cannot go into detail here about how they interpreted RT differences, greater care is needed about how it is described. Perhaps a more neutral/less ambiguous statement might refer to the degree of inhibition or interference from perceiving the distractor.*

Response: The automatic imitation effect is typically interpreted as indexing the degree to which the distractor action is imitated, see Heyes, 2011. We have now changed the phrasing to “*The degree to which the distractor is covertly imitated is captured by the automatic imitation effect”* (lines 32-33) in hopes that this statement is less ambiguous. We believe that a description of the automatic imitation effect as capturing covert imitation – as the measure is intended – is crucial to outline in the Abstract. Facilitatory and inhibitory processes involved are then detailed in the Introduction.

Heyes, C. (2011). Automatic imitation. *Psychological bulletin*, *137*(3), 463.

*Introduction
This is clear and concise. It is stated that the purpose of the online experiment was to replicate the first experiment (page 6, lines 134-136). However, in the Discussion (page 21, lines 436-437), it is stated that the aim was “to validate the appropriateness of conducting speech SRC research online”. Both reasons are worthy motivations, so the latter reason should be highlighted also in the Introduction, especially because online RT experiments are becoming more common. Of particular note are that the experiments in the present study included video stimuli, which are currently quite uncommon in online RT experiments.*

Response: Both motivations are now included in the introduction, lines 148-150: “*Experiment 2 replicated Experiment 1 online, to strengthen our findings through replication (Schmidt, 2009), and to validate that speech SRC tasks can successfully be conducted online (Wilt et al., 2022).”*.

*Methods (Experiments 1 & 2)*

*Page 7, line 144-145 – Typo here: “did not following task instructions” -> “did not follow task instructions”.*

Response: Corrected.

*As shown in Table 1 (page 8), visual articulation and audio occurred at different latencies across the four videos. Although it’s probably unrealistic to keep these timings constant across stimuli (without distorting them), might this affect RTs? Similarly, some responses may take longer to produce compared to others (regardless of whether the response is regarded as native or non-native). Did the authors consider controlling for some of the variability in RTs due to these two factors? While these factors are not of interest, they were intentionally manipulated by the experimenters and could potentially have some influence on the estimates for the effects which are of interest (Compatibility, SOA, Nativeness).*

Response: When considering our stimuli, we had to prioritise certain features over others. E.g., sounds had to be producible by native English speakers, distinguishable from one another visually and auditorily, while ensuring both sounds shared their place of articulation with a native counterpart to minimise the articulatory distance between incompatible pairs. Consequently, some features could not be exactly matched, e.g., alignment of the audio/video. We chose to align prompt timings to visual articulation onset rather than to audio onset, so that the speech sound in each distractor video would be recognisable/distinguishable at the same time. Regarding non-native responses taking longer to produce than native responses, this is captured by the inclusion of the random slope Nativeness (for Experiments 1-2) or Response (for Experiment 3) in the statistical model. Further, Experiment 3 demonstrates that automatic imitation is enhanced for non-native speech distractors regardless of response nativeness, i.e., the effect is still present when producing native responses. We did not control for response RTs beyond native vs. non-native differences, as these cannot be separated form effects of distractor syllable (e.g., and incompatible trial with a “ba” response always occurred with a “la” distractor in Experiments 1-2, hence differences in RTs could be related to the response or to the distractor).

*Page 9, paragraph 1 – Did the speaker provide oral instructions in the video itself or were these instructions written?*

Response: The instructions were provided orally by the speaker in the videos. This is now explicitly stated in the Methods lines 201-202 *“an oral description of how to produce the sound”.*

*Results (Experiments 1 & 2)*

*It is a little confusing that the estimated RT differences in the model summaries (Table 2 and Table 4) do not provide estimates corresponding to the reported raw RTs/RT differences in the text. This is due to the use of dummy (treatment) coding for the models’ predictors, which do not seem to reflect the RT differences of most interest to the authors. I suggest that the authors either provide a brief description of the why the current coding scheme was used or recode the predictors to yield contrasts of interest. The authors may wish to visit this site for general info about coding schemes:* [*https://eur01.safelinks.protection.outlook.com/?url=https%3A%2F%2Fstats.oarc.ucla.edu%2Fr%2Flibrary%2Fr-library-contrast-coding-systems-for-categorical-variables%2F&data=05%7C01%7Channah.wilt.15%40ucl.ac.uk%7Cdd5d426b3b0b4883ca4708db5e146745%7C1faf88fea9984c5b93c9210a11d9a5c2%7C0%7C0%7C638207213941913369%7CUnknown%7CTWFpbGZsb3d8eyJWIjoiMC4wLjAwMDAiLCJQIjoiV2luMzIiLCJBTiI6Ik1haWwiLCJXVCI6Mn0%3D%7C3000%7C%7C%7C&sdata=zj3UZ06j%2Fd22ZweY9x7rLgg48eHXcid2Mpwl5vUXKnQ%3D&reserved=0*](https://eur01.safelinks.protection.outlook.com/?url=https%3A%2F%2Fstats.oarc.ucla.edu%2Fr%2Flibrary%2Fr-library-contrast-coding-systems-for-categorical-variables%2F&data=05%7C01%7Channah.wilt.15%40ucl.ac.uk%7Cdd5d426b3b0b4883ca4708db5e146745%7C1faf88fea9984c5b93c9210a11d9a5c2%7C0%7C0%7C638207213941913369%7CUnknown%7CTWFpbGZsb3d8eyJWIjoiMC4wLjAwMDAiLCJQIjoiV2luMzIiLCJBTiI6Ik1haWwiLCJXVCI6Mn0%3D%7C3000%7C%7C%7C&sdata=zj3UZ06j%2Fd22ZweY9x7rLgg48eHXcid2Mpwl5vUXKnQ%3D&reserved=0)*. In order for the model summaries to show effects like those reported in the text, I suggest the following. The levels of Compatibility and Nativeness can be recoded to -0.5 and 0.5, while backwards difference coding can be used for SOA (see the link above). The model intercept will then be an estimate of the average RT across all Compatibility, Nativeness and SOA conditions, while the effects (and interactions) for Compatibility and Nativeness will be estimates of the RT difference averaged over the levels of the other predictors, and the SOA effects (and interactions) will mirror those presented in the text (i.e., SOA1 vs. SOA2, SOA2 vs. SOA3).*

Responses: The data for all experiments has now been re-analysed using the procedures suggested by the reviewer.

*Although space is limited, are the authors able to provide some more insight into how responses were scored as correct/incorrect? In the non-native task, for example, did the experimenters expect that participants to successfully produce bilabial trills and alveolar lateral fricatives when requested?*

Response: We hope that the following changes/additions make this clearer:

Methods-Procedure, lines 220-222: *“Participants could play the [production instruction] videos as many times as they wanted and were asked to produce each sound at least five times and/or until the researcher was satisfied with their production.”*

Methods-Data processing, lines 238-241: *“For the non-native sounds, productions were considered erroneous if they could not be clearly auditorily identified as attempts to produce either [ʙɑ] or [ɮɑ], and if the spectrogram did not show clear turbulence (for [ʙɑ] and [ɮɑ]) and/or at least one periodic fold (for [ʙɑ] only)”.*

*The placement of the prompt appears to be different in the two experiments (above the video or within the video). Was there any reason for that?*

Response: The placement of the prompt was identical across the three experiments, appearing over the speaker’s lips. In Experiment 1, symbol prompts appeared above the videos for the *instructions* only, for participants to learn the prompt-response pairings. In the SRC task, the symbol always appeared over the speaker’s lips.

*General Discussion*

*Page 22, lines 447-450. The authors report past compatibility effects ranged from 13 ms to 42 ms, one of which is the study by Galantucci et al. (2009). However, the (significant) compatibility effect in the third experiment of that study was just 4 ms.*

Response: In Galantucci et al.’s third experiment, participants produced the same syllable throughout the trials in each task and thus performed what is referred to as a simple response task, vs. a choice response task in our experiments. Simple response tasks lead to smaller automatic imitation effects than choice response tasks (Cracco et al., 2018), hence we cannot compare effects from our study to that of experiments using simple response paradigms. Automatic imitation effect reported in Galantucci et al.’s Experiments 1-2, utilising a response choice paradigm, fall within the 13-42ms range. We have now made this clear, lines 641-645: “*The compatibility effect for native sounds conforms with effects reported in previous SRC speech studies using choice response paradigms with native consonants, ranging from 13-42ms (Kerzel & Bekkering, 2000, Experiments 1-2; Galantucci et al., 2009, Experiments 1-2; Jarick & Jones, 2009; Wilt et al., 2022; Wu et al., 2019).”*

Cracco, E., Bardi, L., Desmet, C., Genschow, O., Rigoni, D., De Coster, L., ... & Brass, M. (2018). Automatic imitation: A meta-analysis. *Psychological Bulletin*, *144*(5), 453.

*The discussion contrasting speech with non-speech actions is interesting. As the authors point out, the task involved individuals producing and perceiving unfamiliar/non-native sounds. Maybe the authors can point out (earlier in the paper when discussing results) that there was a clear effect on responses because the non-native syllables resulted in longer RTs regardless of distractor-related effects (Compatibility, SOA). In other words, the effect of Nativeness provides some evidence that there was a greater burden on processing the non-native responses compared to the native ones.*

Response: This point is now explicitly made in the discussion, lines 636-638: *“RTs were slower in the non-native task by 73ms, reflecting the challenge elicited by producing unfamiliar actions.”*

*Figures/Tables
Fig. 2 and Fig.4 – There is a large amount of space at the top of these figures (approx. RTs >1250 ms), which doesn’t appear to serve any purposes. The captions for both figures say “points in the background show the raw mean RTs for each participant”, though these points aren’t visible to me. Finally, the order of compatible and incompatible conditions is not the consistent across the two figures.*

Response: The space at the top of the figures comprised points representing individual means, which did not appear in the online manuscript despite being in the original document. We have now uploaded the figure as separate files and hope that the points are now visible.

*Table 2 and Table 4 – Do we need the decimals for the estimated mean and SE ms RT values? Why not round them to the closest ms (none of the effects were < 1 ms anyway)?*

Response: The values in the tables are now rounded to the closest ms.

*Reviewer: 3*

*Comments to the Author*

*The authors build on previous research showing that perceived speech is covertly imitated. They ask whether covert imitation differs when the speaker processes familiar (native) sounds versus non-native sounds. Two experiments are conducted in which participants produce a syllable while seeing a video of someone producing either the same or a different distractor sound. The dependent measure is the time to start producing the syllable. Results show that different distractor sounds lead to longer response times. Crucially, this interference effect is larger for non-native than for native sounds.*

*The paper is clear and nicely written. I was not familiar with this literature but the authors did a nice job in introducing all the relevant concepts such that I was able to follow easily.

1/I have one major concern with this work. Unfortunately, I don’t think that the authors can address this concern with the data at hand.  The issue addressed in the paper is whether covert imitation depends on the familiarity with the perceived stimuli (native vs nonnative). I was expecting a design in which only the familiarity with the perceived stimuli was manipulated while the produced stimuli stayed the same across conditions. This was not the case.  In the non-native task, participants produce a non-native sound and the distractor is the same or another nonnative sound. In the native task, the participants produce a native sound and the distractor is the same or another native sound. As a result, it is impossible to know whether the observed difference between the two conditions is due to the difference in the perceived stimuli or in the produced stimuli. The authors do address this caveat in the last paragraph before the conclusion and acknowledge that this is a limitation of the current study. This came too late, as soon as I read the design, I started wondering about this issue and I suspect that many readers will do the same. I don't think that the design is appropriate to address the issue at hand.*

Response: We have now included a third experiment which accounts for the production confound in the original Experiments 1-2, by intermixing native and non-native responses and distractors within a single SRC task.

*2/ In the stimulus-response compatibility task paradigm, participants produce a syllable in the context of a distractor which can either be the same sound or a different sound. The distractor can be presented in the auditory modality only or with a video. Participants are slower to initiate a syllable when the distractor is a different as opposed to the same sound. According to the literature review, this interference effect is interpreted as follows: upon hearing the sound, the speaker covertly simulates the vocal tract actions required to produce that sound (s/he covertly imitates the motor actions required to produce that sound). I am not at all familiar with this literature but I can’t help but wonder whether this is the only possible interpretation. Could it be that processing a distractor activates the corresponding phonological target and that this target can therefore be selected more quickly for production in the “same condition” or that this phonological target must be inhibited in the “different” condition to allow the selection of another phonological syllable? Here no imitation of motor gestures would be involved.*

Response: To determine whether SRC effects truly reflect interference occurring at the response level and are not entirely stimulus related, Jarick and Jones (2009) ran an experiment combining speech distractors and manual responses. The authors did not report a compatibility effect in this paradigm. If the reviewer is interested in reading further into this issue, Ramsey (2018) propose a critical evaluation of automatic imitation effects, to which Cracco and Brass (2019) offer a critical response.

Cracco, E., & Brass, M. (2019). Reaction time indices of automatic imitation measure imitative response tendencies. *Consciousness and cognition*, *68*, 115-118.

Jarick, M., & Jones, J. A. (2009). Effects of seeing and hearing speech on speech production: A response time study. *Experimental brain research*, *195*, 175-182.

Ramsey, R. (2018). What are reaction time indices of automatic imitation measuring?. *Consciousness and cognition*, *65*, 240-254.

*Moreover, in the present study, the distractor is a video. Could it be that seeing an articulatory gesture activates a representation of that gesture? Would that count as covert imitation?
I understand that the authors simply use a paradigm that has been used by others, and that their interpretation of the interference effect follows that provided by other authors before them. Still, I was not convinced and I think that the potential limitations of the paradigm as well as alternative interpretations of the findings should be discussed.*

Response: Speech SRC studies have reported compatibility effects using audiovisual distractors, but also video-only and audio-only distractors. The studies cited in the present manuscript which utilised audio-only distractors are: Galantucci et al., 2009; Ghaffarvand Mokari et al., 2020, 2021; Jarick and Jones, 2009; Roon and Gafos, 2015; Wilt et al., 2022. All reported significant automatic imitation effects.

Galantucci, B., Fowler, C. A., & Goldstein, L. (2009). Perceptuomotor compatibility effects in speech. *Attention, Perception, & Psychophysics*, *71*(5), 1138-1149.

Ghaffarvand Mokari, P., Gafos, A., & Williams, D. (2020). Perceptuomotor compatibility effects in vowels: Beyond phonemic identity. *Attention, Perception, & Psychophysics*, *82*, 2751-2764.

Ghaffarvand Mokari, P., Gafos, A., & Williams, D. (2021). Perceptuomotor compatibility effects in vowels: Effects of consonantal context and acoustic proximity of response and distractor. *JASA Express Letters*, *1*(1).

Jarick, M., & Jones, J. A. (2009). Effects of seeing and hearing speech on speech production: A response time study. *Experimental brain research*, *195*, 175-182.

Roon, K. D., & Gafos, A. I. (2015). Perceptuo-motor effects of response-distractor compatibility in speech: Beyond phonemic identity. *Psychonomic bulletin & review*, *22*, 242-250.

Wilt, H., Wu, Y., Trotter, A., & Adank, P. (2022). Automatic imitation of human and computer-generated vocal stimuli. *Psychonomic Bulletin & Review*, 1-10.

*3/Theoretical background. The authors start the introduction with the claim that seeing and/or hearing speech engages speech production mechanisms. I am not entirely familiar with this literature. Is this a widely shared assumption? Are there no alternative views?*

Response: The activation of production areas in speech perception is now a widely shared assumption. An extensive literature in support of this phenomenon (fMRI, TMS, EEG) is reported in the Introduction, e.g. lines 52-58, lines 63-71.

*4/Statistical analyses*

*-       The authors start with a model that has three factors and their interactions (compatibility, naiveness and SOA). I have several comments here. (a) I don’t think the SOA manipulation is introduced. Why use different SOAS and most importantly, what are the predictions with regard to this manipulation and its interactions with other variables?*

Response: The use of SOA was adopted as it is common practice in SRC experiments. This is now clarified in lines 186-187: “*The utilisation multiple SOAs is standard practice in SRC studies to examine the time course of effects.”*

*(b) I did not understand why the authors decided to remove predictors that did not contribute to the fit. Either the predictors or interactions are included because they address a research question or need to be controlled for (in which case they should be kept in the model irrespective of the fit) or they do not and should not be included in the first place*

Response: The standard practice in our lab is to utilise backward selection to obtain the best fitting model, and hence the most accurate estimates (cf. pre-registered studies Wilt et al., 2022; Wilt, et al., 2022, preprint). As we know there is no universally agreed upon model selection procedure, we have made sure to include all the saturated models in the Appendix for full transparency. We have also described our modelling selection procedure in detail to allow for replicability.

Wilt, H., Wu, Y., Trotter, A., & Adank, P. (2022). Automatic imitation of human and computer-generated vocal stimuli. *Psychonomic Bulletin & Review*, 1-10.

Wilt, H., Wu, Y., & Trotter, A. (2022). Effects of Bottom-up and Top-down Factors on Automatic Imitation. [pre-print]

*-       Information about how the contrasts were set is missing. Looking at the table of results on p. 13, it looks like treatment contrasts were used. Is there a reason to compare SOA2 and SOA3 to SOA1 (rather than say SOA2 and SOA3)? A consequence of using treatment contrasts is that the interaction terms are not interaction terms but simple effects. The discussion of these terms in the paper is therefore incorrect. Centered contrasts should be used (e.g., sum contrasts)*

Response: We have now reanalysed the dataset using centred contrasts and backwards difference coding, following input from the reviewers.

*5/ Preprocessing. Given that the study was not pre-registered, it would be useful to know whether decisions (such as the removal of outliers, the choice of the statistical model) were taken before conducting the statistical analyses.*

Response: Decisions related to data processing and the choice of the statistical model were taken before conducting the analyses, and in line with the standard in our lab, c.f. pre-registered studies Wilt et al. (2022) and Wilt et al. (2022, preprint). Unfortunately, we did not pre-register Experiments 1-2 as these were run before and at the beginning of the COVID-19 pandemic, and our lab did not adopt pre-registration as a common practice until further into the pandemic. Experiment 3 in the current manuscript was pre-registered (AsPredicted #112675). Any changes made to the analyses in the revised manuscript (e.g., use of contrast coding) were made following reviewer comments.

Wilt, H., Wu, Y., Trotter, A., & Adank, P. (2022). Automatic imitation of human and computer-generated vocal stimuli. *Psychonomic Bulletin & Review*, 1-10.

Wilt, H., Wu, Y., & Trotter, A. (2022). Effects of Bottom-up and Top-down Factors on Automatic Imitation.

*Additional comments*

*-In the Discussion the authors repeat the results in detail. I don’t think this is necessary*

Response: Corrected.

*- the terms “Compatible” and “Congruent” are both used to refer to the compatible target-distractor stimuli. I suggest picking one of these terms and sticking to it*

Response: Changed all to “compatible”.

*- Was the order of native and nonnative tasks still balanced after the removal of participants?*

Response: The order of native and non-native tasks was no longer balanced after removal of participants for Experiments 1-2. We ran checks by conducting the analyses with Order and Order x Compatibility as factors and found no effects, hence task order should not be an issue.

*- What is the acoustic and articulatory distance between the two native and the two non native sounds? Is it equivalent? Does this matter?*

Response: Considering the nature of the SRC task, articulatory distance is the primary concern here. This distance was matched as closely as possible for the native and non-native sounds by utilising the same place of articulation for the sound pairs (bilabial vs. alveolar), and utilising only voiced sounds.

REFERENCES

Cracco, E., Bardi, L., Desmet, C., Genschow, O., Rigoni, D., De Coster, L., Radkova, I., Deschrijver, E., & Brass, M. (2018). Automatic imitation: A meta-analysis. *Psychological Bulletin*, *144*(5), 453–500. https://doi.org/10.1037/bul0000143

Cracco, E., & Brass, M. (2019). Reaction time indices of automatic imitation measure imitative response tendencies. *Consciousness and Cognition*, *68*, 115–118.

Galantucci, B., Fowler, C. A., & Goldstein, L. (2009). Perceptuomotor compatibility effects in speech. *Attention, Perception, & Psychophysics*, *71*(5), 1138–1149.

Ghaffarvand Mokari, P., Gafos, A., & Williams, D. (2020). Perceptuomotor compatibility effects in vowels: Beyond phonemic identity. *Attention, Perception, & Psychophysics*, *82*, 2751–2764.

Ghaffarvand Mokari, P., Gafos, A., & Williams, D. (2021). Perceptuomotor compatibility effects in vowels: Effects of consonantal context and acoustic proximity of response and distractor. *JASA Express Letters*, *1*(1), 015204. https://doi.org/10.1121/10.0003039

Heyes, C. (2011). Automatic imitation. *Psychological Bulletin*, *137*(3), 463.

Jarick, M., & Jones, J. A. (2009). Effects of seeing and hearing speech on speech production: A response time study. *Experimental Brain Research*, *195*(2), 175.

Ramsey, R. (2018). What are reaction time indices of automatic imitation measuring? *Consciousness and Cognition*, *65*, 240–254. https://doi.org/10.1016/j.concog.2018.08.006

Roon, K. D., & Gafos, A. I. (2015). Perceptuo-motor effects of response-distractor compatibility in speech: Beyond phonemic identity. *Psychonomic Bulletin & Review*, *22*(1), 242–250. https://doi.org/10.3758/s13423-014-0666-6

Schmidt, S. (2009). Shall We Really Do It Again? The Powerful Concept of Replication Is Neglected in the Social Sciences. *Review of General Psychology*, *13*, 90–100. https://doi.org/10.1037/a0015108

Wilt, H., Wu, Y., & Trotter, A. (2022). *Effects of Bottom-up and Top-down Factors on Automatic Imitation*.

Wilt, H., Wu, Y., Trotter, A., & Adank, P. (2022). Automatic imitation of human and computer-generated vocal stimuli. *Psychonomic Bulletin & Review*. https://doi.org/10.3758/s13423-022-02218-6
